# Description of the dataset on alkoxycarbonylation catalyzed by supported palladium phosphide nanoparticles

**DOI:** 10.1016/j.dib.2026.112995

**Published:** 2026-06-20

**Authors:** Arjun Neyyathala, Felix Jung, Claus Feldmann, Simon Barth, Jan-Dierk Grunwaldt, Ivana Jevtovikj, Stephan A. Schunk, Paolo Dolcet, Silvia Gross, Schirin Hanf

**Affiliations:** a Institute for Inorganic Chemistry, Karlsruhe Institute of Technology, Engesserstr. 15, 76131 Karlsruhe, Germany; b Institute for Chemical Technology and Polymer Chemistry, Karlsruhe Institute of Technology, Engesserstr. 18 / 20, 76131 Karlsruhe, Germany; c Institute of Catalysis Research and Technology, Karlsruhe Institute of Technology, Hermann-von-Helmholtz-Platz 1, 76344 Eggenstein-Leopoldshafen, Germany; d hte GmbH, the high throughput experimentation company, Kurpfalzring 104, 69123 Heidelberg, Germany; e BASF SE, Carl-Bosch Str. 38, 67056 Ludwigshafen, Germany; f Institute of Chemical Technology, Faculty of Chemistry and Mineralogy, Leipzig University, Linnéstr. 3, 04103 Leipzig, Germany; g Department of Chemical Sciences, University of Padova, Via Francesco Marzolo 1, Padova 35131 Italy

**Keywords:** Supported nanoparticles, Heterogeneous catalyst, Element synergy, PXRD, DRIFTS, XPS, STEM

## Abstract

The application of element synergy is a powerful strategy for developing high-performance heterogeneous catalysts. Incorporating a secondary element into the metal lattice can effectively tailor the geometric and electronic properties of surface sites. In particular, the incorporation of phosphorus into metals to form metal phosphides enables the creation of electronically modulated and spatially isolated active sites. This effect is analogous to the effect of ligands on metal centers in homogeneous catalysis, highlighting the potential for designing heterogenized catalysts applying element synergy. In this article, we describe the dataset deposited in the Repo4Cat repository related to the application of supported palladium phosphide nanoparticles in alkoxycarbonylation reactions. The dataset covers the primary data and metadata describing the characterization of the catalyst and application in carbonylation reactions.

Specifications Table

**Table:** 

Subject	Engineering & Materials science
Specific subject area	Heterogeneously catalysed alkoxycarbonylation reaction
Type of data	Figures, tables, primary data and metadata. .txt and .json
Data collection	-Powder X-ray diffraction (PXRD) measurements were performed on a Stoe STADI-MP diffractometer operating with a Ge-monochromatized Cu source (λ=1.54178 Å) in transmission mode with measurements performed until a diffraction angle of 2Ɵ = 90°. -Transmission electron microscopy (TEM), scanning transmission electron microscopy (STEM) and energy dispersive X-ray spectroscopy (EDXS) analysis were performed with a FEI Osiris ChemiSTEM operated at an acceleration voltage of 200 kV. TEM images were obtained and analyzed using a Gatan BM Ultrascan CCD camera and Gatan Digital Micrograph 2.3. - High angle annular dark field-scanning transmission electron microscopy (HAADF-STEM) images were acquired using the software FEI TEM Imaging and Analysis 4.6. -ICP-AES performed using an iCap 6500 device from ThermoFisher Scientific. -X-ray photoelectron spectroscopy (XPS) measurements were performed ThermoScientific Escalab QXi. Spectra were collected employing a monochromatic Al Kα source. -Diffuse reflectance infrared Fourier transform spectroscopy (DRIFTS) measurements were performed using a VERTEX 70 FTIR spectrometer (Bruker) equipped with Praying Mantis diffuse reflection optics (Harrick) and a liquid nitrogen-cooled mercury cadmium telluride detector.
Data source location	Karlsruhe Institute of Technology and University of Padova
Data accessibility	Repository name: Repo4Cat (NFDI4Cat Central Data Repository) Data identification number (handle): https://hdl.handle.net/21.11165/4cat/arp5-s69r } Direct URL to data https://repository.nfdi4cat.org/dataset.xhtml?persistentId=hdl:21.11165/4cat/arp5-s69r
Related research article	Neyyathala A, Jung F, Feldmann C, Barth S, Grunwaldt J-D, Jevtovikj I, Schunk SA, Dolcet P, Gross S, Hanf S. Carbonylation catalysis of aryl halides through active-site engineering. J Catal 2026;456:116,733. https://doi.org/10.1016/j.jcat.2026.116733.

## 1 Value of the Data

• Element synergy is a powerful approach for the development of heterogeneous catalysts. The presented dataset contains information on the characterization of supported palladium phosphide (Pd_3_P) nanoparticles. The shared primary data and metadata can assist researchers in reproducing the synthesis of the catalysts and extending these methods to other catalyst systems based on element synergy principles, enabling the development of supported catalysts.
• The primary data and metadata related to characterizations will help researchers understand the properties of palladium phosphide catalyst and correlate these properties with catalytic activity, thereby supporting the rational catalyst design. Moreover, assists in performing similar analysis techniques and interpreting the data.
• The metadata related to catalyst testing provides guidance for performing reactions under comparable conditions, enabling researchers to benchmark performance against the reported catalyst.
• All datasets are available in machine-readable formats (.json and .txt).

## 2 Background

Various strategies are continuously evolving to design high-performance heterogeneous catalysts, among which element synergy stands out as a key approach [[1], [2], [3]]. This concept involves incorporating a secondary element into the active metal matrix to fine-tune the catalytic sites through electronic and geometric modulation [[4], [5], [6]]. However, identifying the optimal partner element(s) and composition is crucial for enhancing the performance of the base metal, an aspect that highlights the potential of computer-assisted materials screening. Consequently, experimental datasets generated from such studies can play a pivotal role in the discovery of new catalytic materials that exploit element synergy.

The related article to this data article, titled “Carbonylation Catalysis of Aryl Halides through Active-Site Engineering” presents the scientific background, key findings, and methodologies of the study. This data article specifically focussing on providing the description of the primary data and associated metadata files deposited in the Repo4Cat repository, ensuring reproducibility, transparency, and accessibility of the research outcomes. This article serves as a guide for the users to better understand the individual datasets deposited in the repository and to associate them with the related article.

## 3 Data Description

The dataset deposited in the Repo4Cat repository consists of primary and metadata corresponding to catalyst characterization and testing (carbonylation reactions) [7].

Each data files are separately uploaded. For a better understanding, the table below provides description of the catalysts relevant to the dataset (Table 1).

**Table 1:** Catalyst labels used in the work and description.

Catalyst label	Description
Pd_3_P/SiO_2_ (10 wt.% Pd)	Supported Pd_3_P nanoparticles on silica with 10 wt.% Pd loading
Pd_3_P/SiO_2_ (5 wt.% Pd)	Supported Pd_3_P nanoparticles on silica with 5 wt.% Pd loading
Pd_3_P/SiO_2_ (1 wt.% Pd)	Supported Pd_3_P nanoparticles on silica with 1 wt.% Pd loading
Pd/SiO_2_ (5 wt.% Pd)	Supported Pd nanoparticles on silica with 5 wt.% Pd loading (reference catalyst)

The table below provides an overview of the data files deposited on material characterizations such as PXRD, STEM, DRIFTS, CO chemisorption, and XPS. Table 2

**Table 2:** Description of the catalyst characterization data files.

Name of data file	Description	Data format	Data type	Relevant section on related article
PXRD Pd3P on SiO2(5 wt)-raw-2.txt	PXRD measurement data of Pd_3_P/SiO_2_ (5 wt.% Pd)	Text file (.txt)	Primary data	Figure 1a
PXRD Pd3PonSiO2(1 wt)-raw-2.txt	PXRD measurement data of Pd_3_P/SiO_2_ (1 wt.% Pd)	Text file (.txt)	Primary data	Figure S2
PXRD Pd on SiO2 5wt-raw-2.txt	PXRD measurement data of reference catalyst Pd/SiO_2_ (5 wt.% Pd)	Text file (.txt)	Primary data	Figure S1
Pd3PonSiO210wt%.txt	PXRD measurement data of Pd_3_P/SiO_2_ (10 wt.% Pd)	Text file (.txt)	Primary data	Figure S3
PXRD-Meta-2.txt	Details on the PXRD measurements conducted for the catalysts, including the instrument used, sample preparation method, measurement procedure, and data collection parameters	Text file (.txt)	Metadata	Figure 1a, S1, S2, S3
STEM-Meta-2.txt	Details on the HRTEM, STEM, STEM-EDXS measurements conducted for the catalysts, including the instrument used, sample preparation method, measurement procedure, and data collection parameters.	Text file (.txt)	Metadata	Figure 1b, 2, S4, S5, S6
DRIFT-Meta-2.txt	Details of the DRIFTS measurements conducted for the catalysts. Containing information pertaining to the instrument, sample preparation and measurement method	Text file (.txt)	Metadata	Figure 4, S8
DRIFT Pd-100degC-raw-2.txt	DRIFTS measurement data of Pd/SiO_2_ (5 wt.% Pd) catalyst collected at 100 °C	Text file (.txt)	Primary data	Figure S8b
DRIFT Pd-30degC-raw-2.txt	DRIFTS measurement data of Pd/SiO_2_ (5 wt.% Pd) catalyst collected at 30 °C	Text file (.txt)	Primary data	Figure 4b
DRIFT Pd3P-100degC-raw-2.txt	DRIFTS measurement data of Pd_3_P/SiO_2_ (5 wt.% Pd) catalyst collected at 100 °C	Text file (.txt)	Primary data	Figure S8a
DRIFT Pd3P-30degC-raw-2.txt	DRIFTS measurement data of Pd_3_P/SiO_2_ (5 wt.% Pd) catalyst collected at 30 °C	Text file (.txt)	Primary data	Figure 4a
Chemisorption_Pd-raw1–2.txt and Chemisorption_Pd-raw2–2.txt	CO chemisorption measurement data Pd/SiO_2_ (5 wt.% Pd) catalyst	Text file (.txt)	Primary and metadata	TOF estimation
Chemisorption_Pd3P-raw1–2.txt and Chemisorption_Pd3P-raw2–2.txt	CO chemisorption measurement data of Pd_3_P/SiO_2_ (5 wt.% Pd) catalyst	Text file (.txt)	Primary and metadata	TOF estimation
XPS-Pd-Pd.txt	XPS measurement data of Pd 3d signals for Pd/SiO_2_ (5 wt.% Pd) catalyst	Text file (.txt)	Primary and metadata	Figure S7
XPS-Pd3P-P.txt	XPS measurement data of P 2p signals for Pd_3_P/SiO_2_ (5 wt.% Pd) catalyst	Text file (.txt)	Primary and metadata	Figure 3b
XPS-Pd3P-Pd.txt	XPS measurement data of Pd 3d signals for Pd_3_P/SiO_2_ (5 wt.% Pd) catalyst	Text file (.txt)	Primary and metadata	Figure 3a

The catalyst testing data (carbonylation reactions) are provided as separate files, each corresponding to a single test. Every file contains primary data and corresponding metadata. The data on conversion, yield and selectivity are expressed as fractional values (0–1). The following table illustrates the description of the deposited data files and its connection to the related article. Table 3

**Table 3:** Description of catalyst testing data files.

Data file names	Description	Data format	Connection to the related article
A1–2.json, A2–2.json, A3–2.json, A4–2.json, A5–2.json, A6–2.json,	Catalyst testing data for the alkoxycarbonylation of iodobenzene using Pd_3_P/SiO_2_ (5 wt.% Pd) at 100 °C and 6 bar CO pressure in ethanol, with varying reaction times.	JSON (.json)	Table 1, Figure 6
B1–2.json, B2.json, B3.json, B4–2.json	Catalyst testing data for the alkoxycarbonylation of iodobenzene using Pd_3_P/SiO_2_ (5 wt.% Pd) catalyst, applying varying bases.	JSON (.json)	Table 1
C1–2.json, C2–2.json, C3–2.json	Catalyst testing data for the alkoxycarbonylation of iodobenzene with ethanol using Pd/SiO2 (5 wt.% Pd) catalyst.	JSON (.json)	Figure 6, Table 1
k1–2.json, k2–2.json, k3–2.json, k4–2.json, k5–2.json, k6–2.json, k7–2.json, k8–2.json	Catalyst testing data used for the determination of the apparent activation energy for the Pd_3_P/SiO_2_ (5 wt.% Pd) catalyzed alkoxycarbonylation reaction. The experimental results were employed to calculate reaction rates and subsequently the apparent activation energy.	JSON (.json)	Figure 6
S1–2.json, S2–2.json, S3–2.json, S4–2.json, S5–2.json, S6–2.json,	Catalyst testing data for the alkoxycarbonylation with various substrates and nucleophiles using Pd_3_P/SiO_2_ (5 wt.% Pd) catalyst, including phenoxycarbonylation.	JSON (.json)	Table S5
S7–2.json, S8–2.json, S9–2.json	Catalyst testing data for the aminocarbonylation using Pd_3_P/SiO_2_ (5 wt.% Pd) catalyst, with varying temperature.	JSON (.json)	Table S6
Overview Reaction Description-2.txt	Overview of all applied test conditions.	Text file (.txt)	

## 4 Experimental Design, Materials and Methods

The detailed description of the materials and methods used in this work are provide in the related article.

## Limitations

None.

## Ethics Statement

We hereby confirm that we read and follow the ethical requirement of publication in Data in Brief and confirming that this work does not involve human subjects, animal experiments, or any data collected from social media platforms.

## CRediT Author Statement

**Arjun Neyyathala:** Data curation (lead); Investigation (equal); Writing—original draft (equal). **Felix Jung:** Investigation (supporting); Methodology (supporting). **Claus Feldmann:** Validation (equal); Writing—review & editing (supporting). **Simon Barth:** Investigation (supporting); Methodology (supporting). **Jan-Dierk Grunwaldt:** Validation (equal); Writing—review & editing (supporting). **Ivana Jevtovik:** Investigation (supporting); Methodology (supporting). **Stephan A. Schunk:** Conceptualization (equal); Supervision (equal); Writing—review & editing (supporting). **Paolo Dolcet:** Investigation (supporting); Methodology (supporting). **Silvia Gross:** Investigation (supporting); Methodology (supporting). **Schirin Hanf:** Conceptualization (lead); Methodology (equal); Writing—original draft (equal); Writing—review & editing (lead).

## Data Availability

NFDI4Cat Central Data RepositoryDescription of the dataset on alkoxycarbonylation catalyzed by supported palladium phosphide nanoparticles (Original data).

## Appendix Supplementary materials

Image, application 1
