# Supplementary material for: Description of the dataset on alkoxycarbonylation catalyzed by supported palladium phosphide nanoparticles
Source: Data Brief. 2026 Jun 20;67:112995. doi: 10.1016/j.dib.2026.112995 (PMC13316290; doi:10.1016/j.dib.2026.112995)
Supplement: Supplementary file 1 [file mmc1.pdf]

Journal of Catalysis 456 (2026) 116733

Contents lists available at ScienceDirect

Journal of Catalysis

journal homepage: www.elsevier.com/locate/jcat

Research article

Carbonylation catalysis of aryl halides through active-site engineering

Arjun Neyyathala [a], Felix Jung [a], Claus Feldmann [a], Simon Barth [b,c], Jan-Dierk Grunwaldt [b,c], Ivana Jevtovikj [d], Stephan A. Schunk [d,e,f], Paolo Dolcet [b,g], Silvia Gross [b,g], Schirin Hanf [a,*]

[a] Institute for Inorganic Chemistry, Karlsruhe Institute of Technology, Engesserstr. 15, 76131 Karlsruhe, Germany
[b] Institute for Chemical Technology and Polymer Chemistry, Karlsruhe Institute of Technology, Engesserstr. 18 / 20, 76131 Karlsruhe, Germany
[c] Institute of Catalysis Research and Technology, Karlsruhe Institute of Technology, Hermann-von-Helmholtz-Platz 1, 76344 Eggenstein-Leopoldshafen, Germany
[d] hte GmbH, the high throughput experimentation company, Kurpfalzring 104, 69123 Heidelberg, Germany
[e] BASF SE, Carl-Bosch Str. 38, 67056 Ludwigshafen, Germany
[f] Institute of Chemical Technology, Faculty of Chemistry and Mineralogy, Leipzig University, Linnéstr. 3, 04103 Leipzig, Germany
[g] Department of Chemical Sciences, University of Padova, Via Francesco Marzolo 1, Padova 35131, Italy

A R T I C L E I N F O

Keywords:
Alkoxycarbonylation
Heterogeneous catalysis
Palladium phosphide
Supported nanoparticles
Element synergy
Carbonylation of natural synthons

A B S T R A C T

Crystalline palladium phosphide nanoparticles supported on silica (Pd_3P/SiO_2, 5 wt% Pd) are explored as catalysts for the alkoxycarbonylation of lignin-derived aromatic synthons, using model aryl halides as representative substrates. The detailed characterization by PXRD, HAADF-STEM, HRTEM, EDX, ICP-AES, XPS, CO-DRIFTS, and CO chemisorption confirmed the formation of the Pd_3P phase with uniform nanoparticle size distribution. The catalytic performance was evaluated in a three-phase reaction system comprising a CO gas atmosphere, a liquid phase containing the solvent and substrate and a solid catalyst. The incorporation of phosphorus into the palladium lattice resulted in a more than two-fold enhancement in catalytic activity compared to conventional Pd-based heterogeneous catalysts. The Pd_3P/SiO_2 catalyst also outperformed several reported heterogeneous and commonly used homogeneous catalysts. This enhanced reactivity is attributed to the electronic and geometric effects introduced by phosphorus, which generate highly active, spatially-isolated Pd sites. These findings demonstrate the potential of Pd–P phase engineering for the design of the next-generation of carbonylation catalysts.

1. Introduction

In recent years, alternative feedstocks have gained significant attention due to their renewable nature, abundance, and their potential to replace petroleum-based chemicals in a sustainable circular economy. Among these, lignin stands out as a particularly promising candidate. As one of the most abundant biopolymers on Earth, lignin is a major byproduct of the pulp and paper industry and biorefineries. Its high aromatic content makes it an ideal precursor for the production of high-value chemicals through depolymerization and subsequent functionalization reactions. While established methods already convert lignin into oxygen- and heteroatom (N, S, …)-containing aromatics [1–3], recent advances have enabled the direct transformation of lignin into aryl halides [4]. This development is particularly exciting because aryl halides are indispensable synthons with broad applications in life and materials sciences in both academic and industrial settings. Traditionally, their aromatic hydrocarbon precursors are derived from petroleum or coal through catalytic reforming and steam cracking, followed by subsequent functionalization.

The ability to produce lignin-derived aryl halides opens the door to further valuable transformations, most notably carbonylation reactions. These reactions involve the insertion of carbon monoxide into C–X (X = halide) bonds in the presence of a nucleophile (NuH), yielding carboxylic acids, amides, esters, and azides [5–8], all of which are key intermediates for pharmaceuticals, agrochemicals, polymers, and specialty chemicals [6,7,9]. As catalysts, molecular palladium complexes, either pre-synthesized, such as $[PdCl_2(dppp)]$ (dppp = 1,3-bis(diphenylphosphino)propane) [10], $[PdCl_2(dcpp)]$ (dcpp = 1,3-bis(dicyclohexylphosphino)propane) [10], $[PdBrPh(PPh_3)_2]$ [11], $[Pd(PPh_3)_4]$ [12], $[PdCl_2(PCy_3)_2]$ [13], $[Pd(2\text{-}(CH_3NH)Py\text{-}PPh_2)_2](OAc)_2$ [14], or formed *in-situ* during the reaction from palladium-based compounds, e.g. palladium acetate or chloride, in the presence of ligands, such as triphenylphosphine (PPh_3) [15], 1,1′-bis(diphenylphosphino)ferrocene (dppf) [16], 1,3-bis(dicyclohexylphosphino)propane [17],

* Corresponding author.
E-mail address: schirin.hanf@kit.edu (S. Hanf).

https://doi.org/10.1016/j.jcat.2026.116733
Received 21 November 2025; Received in revised form 29 January 2026; Accepted 2 February 2026
Available online 5 February 2026
0021-9517/© 2026 The Author(s). Published by Elsevier Inc. This is an open access article under the CC BY license (http://creativecommons.org/licenses/by/4.0/).

1,3-bis(diisobutylphosphino)propane (DIBPP) [18], 2,2′-bis(diphenylphosphino)-1,1′-binaphthyl (BINAP) [19], (9,9-dimethyl-9H-xanthene-4,5-diyl)bis(diphenylphosphane) (Xantphos) [20], etc. are known to exhibit excellent activities [21]. Even though such homogeneous catalysts exhibit high activity and selectivity, they pose significant challenges in terms of catalyst handling, stability, separation, and reuse. This ultimately may lead to additional separation costs on an industrial scale, a loss of noble metal, product contamination, and, as a consequence, increased overall process costs [5]. One strategy to overcome these challenges is the development of high-performing heterogeneous catalysts, through which a facile catalyst handling and reuse as well as a more efficient product separation becomes possible.

Despite the well-established industrial significance of alkoxycarbonylation reactions, research on palladium-based heterogeneous catalysts remains significantly underexplored compared to the established homogeneous counterparts. This can partly be attributed to the deactivation phenomena based on the aggregation of Pd in the presence of CO in solid catalysts, which leads to the formation of catalytically inactive species, thereby limiting large-scale applications [5,22]. The alkoxycarbonylation of aryl chlorides using Pd supported on activated carbon (Pd/C), as reported by Dufaud *et al.*, is one of the earliest studies in the field of heterogeneously catalyzed carbonylation reactions [22]. Based on these initial findings, in the past two decades many research groups have reported various heterogeneous/heterogenized alkoxycarbonylation catalysts. Some examples are supported palladium particles, such as Pd/C [23], Pd/CeO_2 [24], Pd/Fe_3O_4 [25], palladium supported on N-doped carbon (Pd/PdO@NGr-C) [26], porous organic cage-stabilized Pd nanoparticles (Pd@CC3-R–MeOH) [27], immobilized Pd complexes [28,29] and Pd catalysts in the presence of ionic liquids [8,30]. Despite significant research efforts, most reported catalysts exhibit low activity, limited lifetime, or require complex synthesis procedures. Furthermore, previous studies have rarely focused on the intrinsic catalyst activity, reaction kinetics, or any systematic benchmarking. These limitations underscore the need for (i) facile and sustainable synthesis methods to develop highly active solid-state catalysts, and (ii) a deeper mechanistic understanding of performance-related factors through rigorous kinetic studies in carbonylation chemistry.

A very effective approach for the development of highly active and selective heterogeneous catalysts is the strategic combination of a catalytically active metal with a p-block element, which enables the modulation of the metal centers' electronic and geometric properties [31–33]. Through this so called “d-block-p-block element combination strategy”, highly active ensembles or sites can be formed on the catalyst surface, thereby tuning the adsorption properties of substrates and intermediates [34,35]. In this context, the incorporation of phosphorus or sulfur into metal matrices to form metal phosphides or sulfides has demonstrated exceptional catalytic performance and has been investigated across various catalytic reactions [33,35–39]. Notable examples highlighting the impact of surface motifs, engineered through phosphorus or sulfur incorporation, include the exceptional performance of spatially isolated metal trimers in bulk nickel phosphide catalysts (Ni_2P, Ni_5P_4) [40] and palladium sulfides supported on carbon nitride (Pd_4S/C_3N_4 and Pd_3S/C_3N_4) [34] for the semi-hydrogenation of alkynes. Additionally the enhanced hydroformylation activity driven by Rh_4 tetrameric motifs in supported rhodium sulfides ($Rh_{17}S_{15}/SiO_2$) [35], and the selective tuning of electrocatalytic nitrate reduction reactions enabled by active nickel ensembles in nickel phosphide deposited on carbon (Ni_2P/C) [41] further highlight the significance of these structural modifications. However, not only do the spatial arrangements of the metal centers and the formation of unsaturated metal sites play a key role, but the underlying charge transfer processes between the metal and the p-block element are equally critical. These processes mimic the electronic metal–ligand interactions observed in classical molecular catalysts [32,42].

In this context, metal phosphides are particularly attractive materials, as the variation of the metal-to-phosphorus ratio affords substantial structural diversity, enabling the development of a broad library of highly active and selective catalysts, while also offering high stability under harsh reaction conditions [31,43]. Despite exhibiting excellent catalytic performance, metal phosphides, to the best of our knowledge, have not been reported in the context of alkoxycarbonylation reactions and particularly not for the transformation of renewable synthons. Therefore, in this study, supported palladium phosphide (Pd_3P) nanoparticles were synthesized, characterized and successfully applied in the alkoxycarbonylation of aryl iodides, which can be derived from lignin as sustainable and renewable synthons. The Pd_3P catalysts exhibited superior activity compared to supported Pd nanoparticles and several reported heterogeneous and homogeneous systems. This work does not only highlight the potential of metal phosphides as efficient catalysts for sustainable carbonylation processes but also offers valuable insights into the design of heterogeneous catalysts through the d-block-p-block element combination strategy.

2. Experimental section

2.1. Materials

Palladium acetate ($Pd(OAc)_2$) with a purity ≥99.99 % and 85 wt% phosphoric acid (H_3PO_4) were purchased from Sigma Aldrich and hydrochloric acid (37 %) from Thermo Scientific Chemicals. The support material silica (Cariact, Q 20C) was purchased from Fuji Silysia Chemical Ltd. Triethylamine (purity 99 %) as well as *n*-decane were purchased from abcr and iodobenzene with a purity ≥99 % from TCI. Ethanol with a purity ≥99.8 % was obtained from Carl Roth and acetone (HPLC grade), which was used for the GC sample preparation, was purchased from Fischer Scientific GmbH. All chemicals were used without any further purification. The gases used for this work, hydrogen (purity 99.999 %) and carbon monoxide (purity 98 %) were purchased from Air Liquide.

2.2. Catalyst synthesis

The supported palladium phosphide catalyst was synthesized using a previously reported procedure [44]. In a typical synthesis of Pd_3P/SiO_2 (5 wt% Pd), 100 mg $Pd(OAc)_2$ and 10 μL H_3PO_4 were dissolved in concentrated hydrochloric acid *via* sonication. This solution was then added drop wise to 0.94 g silica (incipient wetness impregnation), which was then dried at 60 °C for 12 h. The dried impregnated material was further thermally treated in pure hydrogen at 400 °C for 8 h, with a heating ramp of 50 °C/min. The thermal treatment of the dried impregnated precursors was carried out in a quartz Schlenk tube, which was equipped with a pressure relief valve. After evacuation of the flask, the hydrogen gas was introduced and subsequently heated to the required temperature using a box furnace. The synthesized catalysts were either used directly in the catalytic experiments or stored in an Ar-filled glovebox (MBraun) prior to subsequent use/analysis.

Supported palladium nanoparticles (Pd/SiO_2, 5 wt% Pd) were synthesized in a similar manner, however without the addition of phosphoric acid.

2.3. Catalyst characterization

Powder X-ray diffraction (PXRD) measurements were performed on a Stoe STADI-MP diffractometer operating with a Ge-monochromatized Cu source ($\lambda = 1.54178$ Å) in transmission mode with measurements performed until a diffraction angle of $2\theta = 90°$. Transmission electron microscopy (TEM), scanning transmission electron microscopy (STEM) and energy dispersive X-ray spectroscopy (EDXS) analysis were performed with a FEI Osiris ChemiSTEM operated at an acceleration voltage of 200 kV. TEM images were obtained and analyzed using a Gatan BM Ultrascan CCD camera and Gatan Digital Micrograph 2.3. High angle annular dark field-scanning transmission electron microscopy (HAADF-

STEM) images were acquired using the software FEI TEM Imaging and Analysis 4.6. In order to determine the particle size distributions of the samples, measurement of 200 nanoparticle diameters per sample was carried out. Elemental mapping with EDXS was performed using a Bruker Super-X EDXS system and quantification of the data was performed in Bruker Esprit 2.3 using the Cliff-Lorimer method with theoretical k factors. In order to prepare the samples for the electron microscopy analysis, the catalysts were dispersed in ethanol and a drop was carefully added to a grid (Cu grids covered by an amorphous carbon film) and subsequently dried at 60 °C overnight. The quantification of Pd in the samples was done *via* inductively coupled plasma-atomic emission spectroscopy (ICP-AES) performed using an iCap 6500 device from ThermoFisher Scientific. The samples for the analysis were dissolved in nitric acid, hydrofluoric acid and hydrochloric acid at 130 °C for 12 h using a digestion system. CO chemisorption measurements were performed with an instrument made in house with an analyzer from Rosemount Xstream. X-ray photoelectron spectroscopy (XPS) measurements were performed with a ThermoScientific Escalab QXi. Spectra were collected employing a monochromatic Al K_α source ($\lambda = 1486.6$ eV), with a spot size of 650 μm and using a flood gun for charge compensation. For the XPS measurements, the catalyst was stored under an inert atmosphere in a glovebox, and immediately transferred to the XPS instrument with minimal exposure to air. The measurements were performed without any additional pre-treatment. High resolution spectra of regions of interest were collected using a pass energy of 25 eV and a step of 0.05 eV. Where not specified, the NIST X-ray Photoelectron Spectroscopy Database (Version 5) was used for speciation of spectral lines [45]. Diffuse reflectance infrared Fourier transform spectroscopy (DRIFTS) measurements were performed using a VERTEX 70 FTIR spectrometer (Bruker) equipped with Praying Mantis diffuse reflection optics (Harrick) and a liquid nitrogen-cooled mercury cadmium telluride detector. The granulated catalyst (50 mg, sieve fraction 100–200 μm) was placed inside a high-temperature *in-situ* cell (Harrick) covered by a CaF_2 window. The gas flow (50 mL/min) was dosed using mass flow controllers (Bronkhorst). After 1 h of inert treatment in argon at 150 °C to remove physically adsorbed water, the catalyst was cooled to 30 °C and exposed to the CO-containing atmosphere (4000 ppm CO and balance Ar) for 60 min. Further, the system was flushed with Ar (50 mL/min) for 60 min, while scans were collected continuously. After the spectra were collected, the temperature was increased to 100 °C and the CO adsorption procedure was repeated. The spectra were collected in diffuse reflectance mode, converted to pseudo absorbance using OriginPro® 2021b. Scans of the pretreated catalyst at the corresponding temperature were used as a background. A linear background correction was applied.

2.4. Catalyst testing

Catalytic tests were performed in a Parr 5500 series compact reactor with 25 mL capacity equipped with an overhead stirrer. In a typical reaction, iodobenzene (1 mmol), triethylamine (3 mmol), *n*-decane (100 μL) and the catalyst (0.5 mol % Pd) were added to ethanol (4 mL) in the reactor. Further the reactor was flushed 3 times with carbon monoxide and pressurized to the required pressure and heated to the reaction temperature. A stirring speed of 1000 rpm was used for all tests to ensure the full saturation of CO in the liquid reaction medium. After the reaction time, the heater was turned off and the reactor was allowed to cool down to ambient conditions. Prior to the opening of the reactor, the remaining gas was slowly released and the product obtained was filtered and analyzed *via* GC–MS analysis.

The tests for the determination of the apparent activation energies were performed with iodobenzene conversions lower than 30 %. In order to determine the turnover frequency of the Pd_3P/SiO_2 (5 wt% Pd) and Pd/SiO_2 (5 wt% Pd) catalysts, the dispersion values obtained from the CO chemisorption measurements were used as a basis and the estimations were done at iodobenzene conversions in the range of 5 and 30 %.

For the recovery and recycling tests of the catalysts, the catalysts were removed after the reaction from the liquid products through centrifugation at 12000 rpm, followed by washing with acetone for 3 times. The washed catalysts were dried at 60 °C overnight and used for the next runs without any further activation steps and without any further thermal treatment. A sufficient number of parallel reactions were carried out to generate the required catalyst amount for subsequent recycling tests.

The carbonylation products obtained from the catalytic tests were analyzed qualitatively and quantitatively *via* gas chromatography-mass spectrometry (GC–MS) with an Agilent model 8860 GC and 5977B MSD. In order to perform a quantitative analysis, *n*-decane was used as internal standard. For a typical analysis, the product obtained from the catalyst testing was filtered using a syringe filter (0.2 μm) and 80 μL samples were added to 1 mL of acetone in a GC vial. The GC–MS device was equipped with a silica capillary column (ID: 0.25 mm, film thickness: 0.25 μm, and length: 30 m). An injection volume of 1 μL, split ratio of 1:100 and inlet temperature of 310 °C was used for the analysis.

3. Results and discussions

3.1. Catalyst preparation and characterization

Silica-supported palladium phosphide nanoparticles with 5 wt% Pd metal loading, Pd_3P/SiO_2 (5 wt% Pd), were synthesized following a reported procedure [44]. Therefore, palladium(II) acetate ($Pd(OAc)_2$) and phosphoric acid (H_3PO_4) with a Pd to P molar ratio of 3:1 were dissolved in hydrochloric acid (HCl) and impregnated on silica as support material. After drying, the impregnated support underwent thermal treatment in pure hydrogen at 400 °C. The temperature for the thermal treatment was selected based on our previous studies to ensure the complete formation of the Pd_3P phase on the silica support [37,44]. The PXRD analysis of the sample confirmed the formation of phase pure Pd_3P on silica (Fig. 1a), and the occurrence of very broad reflexes in the diffractogram indicates the formation of small crystallites of palladium phosphide. Similarly, the PXRD analysis of the reference catalyst, Pd/SiO_2 (5 wt% Pd) was performed, whereby reflexes corresponding to the face-centered cubic structure of palladium were observed (Fig. S1). High resolution transmission electron microscope (HRTEM) analysis performed for Pd_3P/SiO_2 (5 wt% Pd) indicated a lattice fringe distance of 2.0 Å, which aligns with the d spacing of crystalline Pd_3P (122) (Fig. 1b). This finding, in addition to the PXRD analysis, provides conclusive proof for the formation of targeted Pd_3P phase as supported nanoparticles. Furthermore, EDXS analysis combined with STEM imaging showed an uniform distribution of phosphorus and palladium on silica (Fig. 2), with an average Pd/P atomic ratio of 3.03, which is again in line with the atomic ratio of the targeted phase. The palladium loading on silica for the Pd_3P/SiO_2 (5 wt% Pd) catalyst was quantified using ICP-AES, and was determined to be 4.95 wt%.

Spherical shaped particles with an average particle size of 5.6 nm uniformly distributed over the silica support were observed (Fig. 2) through HAADF-STEM imaging of Pd_3P/SiO_2 (5 wt% Pd), whereas slightly bigger particles with an average size of 6.6 nm were identified (Fig. S4) for the pure palladium-based catalyst, Pd/SiO_2 (5 wt% Pd). The difference in particle size of palladium phosphide and metallic palladium nanoparticles can be attributed to the reduced crystallization tendency of the Pd_3P phase [46], which results in the formation of smaller crystallites even at a temperature of 400 °C, where the thermal treatment was carried out and the phase formation took place.

To gain insights into the surface properties of the catalysts and the electronic modulation of Pd induced by phosphorus incorporation, XPS analysis was performed on the Pd_3P/SiO_2 (5 wt% Pd) sample and Pd/SiO_2 (5 wt% Pd) as a reference. Deconvolution of the Pd 3d spectra for Pd_3P/SiO_2 (5 wt% Pd) revealed the formation of palladium phosphide (Fig. 3a). The peak at 335.9 eV corresponds to the Pd $3d_{5/2}$ signal of

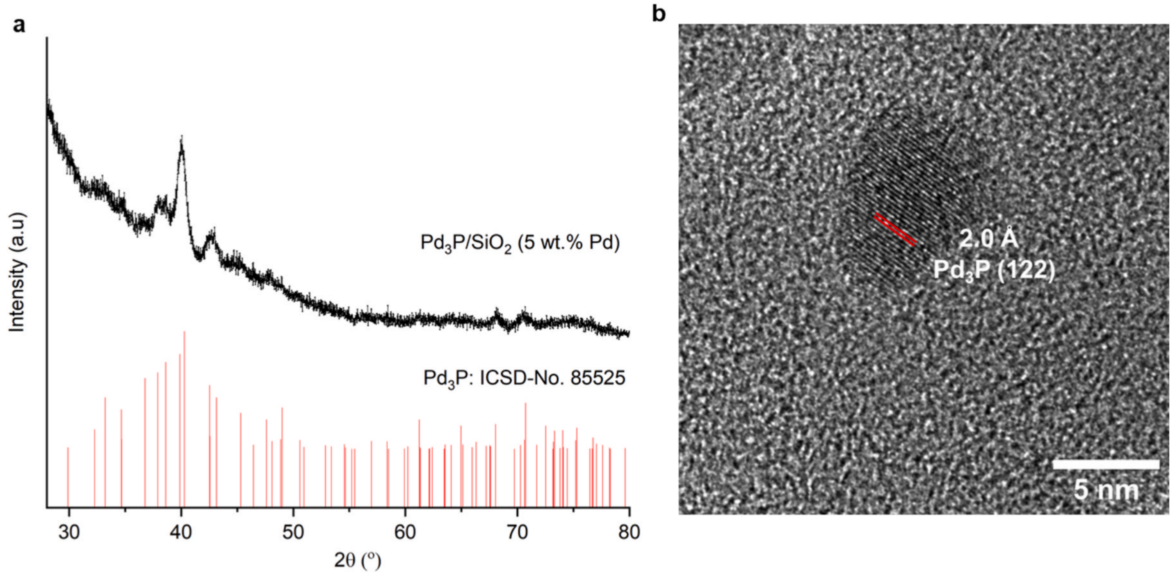

Fig. 1. Phase identification of Pd_3P/SiO_2 (5 wt% Pd) through a) PXRD and b) HRTEM lattice fringe-based assessment. (Pd_3P: ICSD-No. 85525).

palladium with slightly cationic character, in line with a $Pd^{\delta+}$ species, and accounting to the charge transfer from Pd to P, while the peak at around 337.1 eV can be assigned to Pd^{2+} species [47–49]. The contribution of the peak corresponding to slightly cationic Pd species is much higher than that of the Pd^{2+} species, which is in line with the reported XPS spectra of other supported palladium phosphide catalysts [47]. Furthermore, an additional peak centered around 338.0 eV, with relatively low intensity, appears in the Pd_3P/SiO_2 (5 wt% Pd) spectrum, indicating the presence of Pd species with a higher positive oxidation state than Pd^{2+}. This feature can be tentatively attributed to Pd^{4+} species, which are characteristic of supported palladium-based catalysts [50–52]. The P 2p region of Pd_3P/SiO_2 (5 wt% Pd) partially overlaps with the Si 2p plasmon signal (see the broad feature in Fig. 3b), but careful deconvolution reveals two distinct phosphorus species (Fig. 3b). The feature at 130.5 eV can be assigned to the P $2p_{3/2}$ signal of phosphorus bonded to Pd, consistent with the occurrence of palladium phosphide [47]. Additionally, the peak at a higher binding energy of 135.1 eV, can be assigned to surface phosphate species (Si–O–P linkage), resulting from the interaction of phosphorus species with the silica support [47,53]. The XPS analysis of the Pd/SiO_2 (5 wt% Pd) reference catalyst shows the coexistence of Pd^0, Pd^{2+} and Pd^{4+}species (Fig. S7), with corresponding Pd $3d_{5/2}$ signals at 335.3 eV, 336.6 eV and 338.7 eV respectively, consistent with the results reported for other supported Pd catalysts [50–52,54]..

For the determination of the metal dispersion of the catalysts, CO chemisorption measurements were performed. A slightly reduced metal dispersion of 8.5 % was observed for Pd_3P/SiO_2 (5 wt% Pd) in comparison to 9.9 % for Pd/SiO_2 (5 wt% Pd). This observation may be attributed to the reduced CO affinity of Pd_3P surfaces compared to pure Pd. However, this finding could also suggest the possibility of residual phosphorus species on the surface of Pd_3P, likely resulting from the thermal treatment being conducted under stagnant conditions.

Additional insight into the surface properties of the supported Pd_3P and Pd catalysts was obtained *via in-situ* DRIFTS measurements with CO as a probe molecule (Fig. 4 and Fig. S8). Upon CO exposure at 30 °C, *in-situ* DRIFTS spectra of both Pd_3P/SiO_2 (5 wt% Pd, Fig. 4a) and Pd/SiO_2 (5 wt% Pd, Fig. 4b) exhibited a prominent absorption band at ∼ 2092 cm^{-1}, characteristic of linearly adsorbed CO on well-coordinated Pd^0 terrace sites [55–58]. A shoulder around ∼2057 cm^{-1} was also observed for both catalysts and is attributed to CO bound to Pd^0 atoms in undercoordinated environments, such as edge and step sites [55–57]. Notably, Pd_3P/SiO_2 displayed a relatively more pronounced 2057 cm^{-1} shoulder, suggesting a higher proportion of undercoordinated Pd species compared to Pd/SiO_2.

Due to the overall low signal intensity and contributions from gas-phase H_2O vibrational modes in the optical path, a reliable quantification of the ratio between fully coordinated and undercoordinated Pd sites was not feasible. Additionally, a weak band near 2130 cm^{-1} was observed for both catalysts, which is typically associated with CO bound to Pd^{2+} species [55,56,58]. Despite expectations based on the XPS results and previous studies,[41] the anticipated blue shift indicative of cationic Pd species in the Pd_3P phase was not clearly resolved, likely due to the limited signal-to-noise ratio [35,42].

Beyond the differences between individual species in Pd_3P/SiO_2 and Pd/SiO_2, notable variations in CO affinity were observed. Pd/SiO_2 (5 wt %) exhibited a significantly more intense CO adsorption signal at 30 °C compared to Pd_3P/SiO_2 (5 wt% Pd), indicating a stronger CO adsorption on the pure Pd catalyst (Fig. 4b). This contrast became even more pronounced at 100 °C, where no CO adsorption was detected on Pd_3P, while Pd still showed characteristic Pd–CO absorption bands (Fig. S6), although less intense than at 30 °C. This trend is consistent with the reported CO chemisorption measurements. The reduced CO adsorption affinity of Pd_3P can be attributed to an electron transfer from Pd to P in the phosphide phase, which reduces the electron density on Pd, as observed in the Pd 3d XP spectrum of Pd_3P/SiO_2 [49,59]. As a consequence, the ability of Pd in Pd_3P to engage in π* back-donation to CO is diminished, which leads to a weaker CO adsorption [42].

3.2. *Alkoxycarbonylation reactions*

The synthesized supported palladium phosphide catalyst, Pd_3P/SiO_2 (5 wt% Pd) was applied in the alkoxycarbonylation reaction of iodobenzene. The reaction system comprises CO in the gas phase, iodobenzene dissolved in a solvent as the liquid phase, and a solid catalyst, thereby constituting a three-phase system. Initial experiments were performed at 100 °C with CO pressure of 6 bar, 3 equivalents (in comparison to iodobenzene) of triethylamine (Et_3N) as base, and ethanol as the solvent and nucleophile (NuH) in batch mode. Under these applied reaction conditions, an iodobenzene conversion of 97 % with 100 % selectivity to ethyl benzoate (Table 1, entry 1) was observed. A complete conversion was achieved by extending the reaction time to 3 h (Table 1, entry 2). This provides an initial indication that the active catalyst synthesis strategy, based on the d-block-p-block element combination, which has been successfully applied in hydroformylation [35,36],

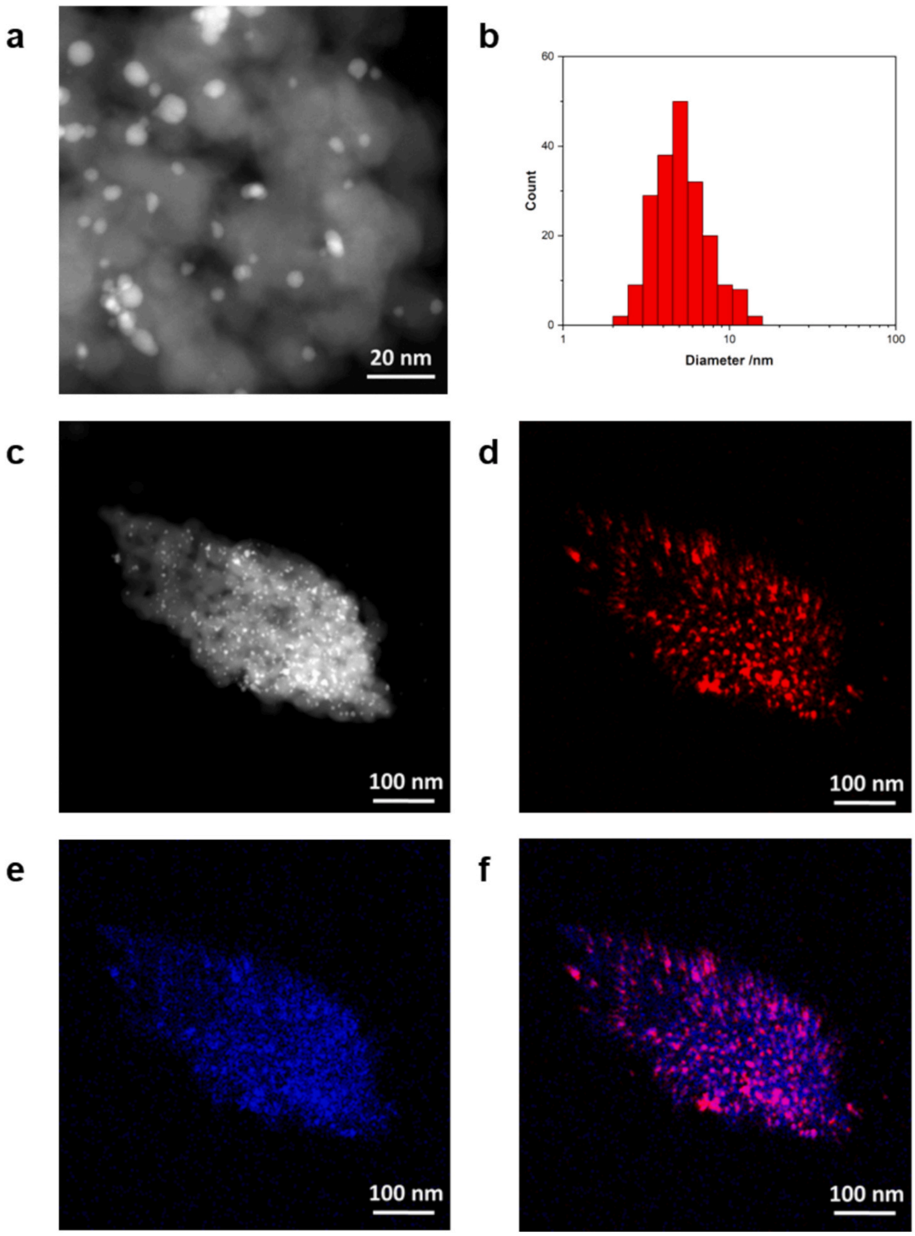

Fig. 2. Scanning transmission electron microscopy images of Pd_3P/SiO_2 (5 wt% Pd) with a) HAADF-STEM showing the distribution of spherical palladium phosphide nanoparticles on silica, b) the particle size distribution histogram showing the average particle size of 5.6 ± 2.4 nm, c) a HAADF-STEM image for the EDXS mapping of d) palladium, e) phosphorus, and f) combined Pd and P (Pd Kα: red, P Kα: blue). (For interpretation of the references to colour in this figure legend, the reader is referred to the web version of this article.)

hydrogenation [34], and electrocatalysis [41], can also be effectively translated to alkoxycarbonylation chemistry.

To investigate the role of the base in the alkoxycarbonylation reaction, various bases, including NaOAc, KOH, and K_2CO_3, were screened to evaluate their impact on the catalytic activity and identify the most suitable base for further studies. By performing the reaction with Et_3N, a 97 % conversion of iodobenzene could be achieved (Table 1, entry 1), whereas the use of NaOAc, KOH, and K_2CO_3 only resulted in lower conversions of 40 %, 70 % and 56 %, respectively (Table 1, entries 3, 4, and 5). In analogy to homogeneous catalyst systems, we conclude that the advantage is the superior solubility of triethylamine in the reaction medium in comparison to the other bases. Similar trends, where Et_3N outperforms other bases, have been reported in previous studies on heterogeneously catalyzed alkoxycarbonylation reactions [60,61]. Moreover, the use of strong inorganic bases, such as K_2CO_3 and KOH, led to the formation of small amounts of benzene as unwanted side product *via* a reductive deiodination pathway (Table 1, entry 4 and 5) [62].

Further, a control experiment was conducted in the absence of base to assess its critical role in the reaction. A significantly reduced iodobenzene conversion of only 7 % to ethyl benzoate was observed after 2 h reaction time (Table 1, entry 6). We attribute the necessity of a base in this reaction to the reaction mechanism of the alkoxycarbonylation of aryl halides (Fig. S9). The base facilitates the decomposition of the acyl-palladium complex ($[ArCO(L_n)PdX]$) by promoting ester formation through the alcohol, while simultaneously neutralizing the generated HX. This process is crucial for regenerating the active Pd site, which can then enter the next catalytic cycle [5,7,13,63,64]. This highlights the requirement of a base during the reaction to complete the catalytic cycle, enhance the reaction rate, and achieve higher conversion levels. Considering these aspects and the performance of various applied bases (Table 1, entries 1, 3, 4 and 5), all subsequent catalytic reactions were performed with Et_3N.

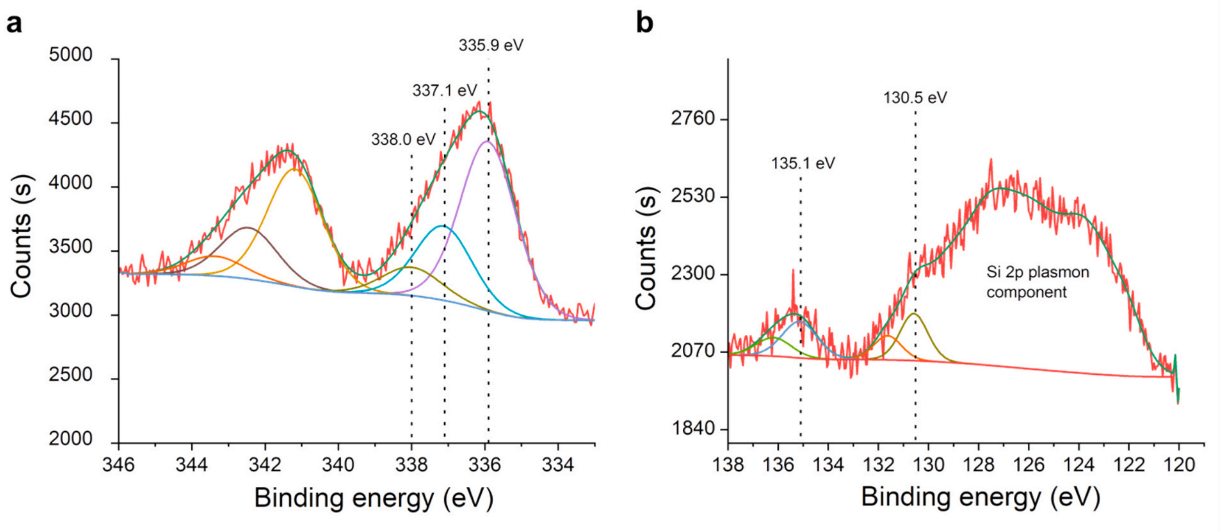

Fig. 3. XPS analysis of Pd_3P/SiO_2 (5 wt% Pd) for a) Pd 3d and b) P 2p. The XPS measurements were carried out on catalysts without any further pre-treatment.

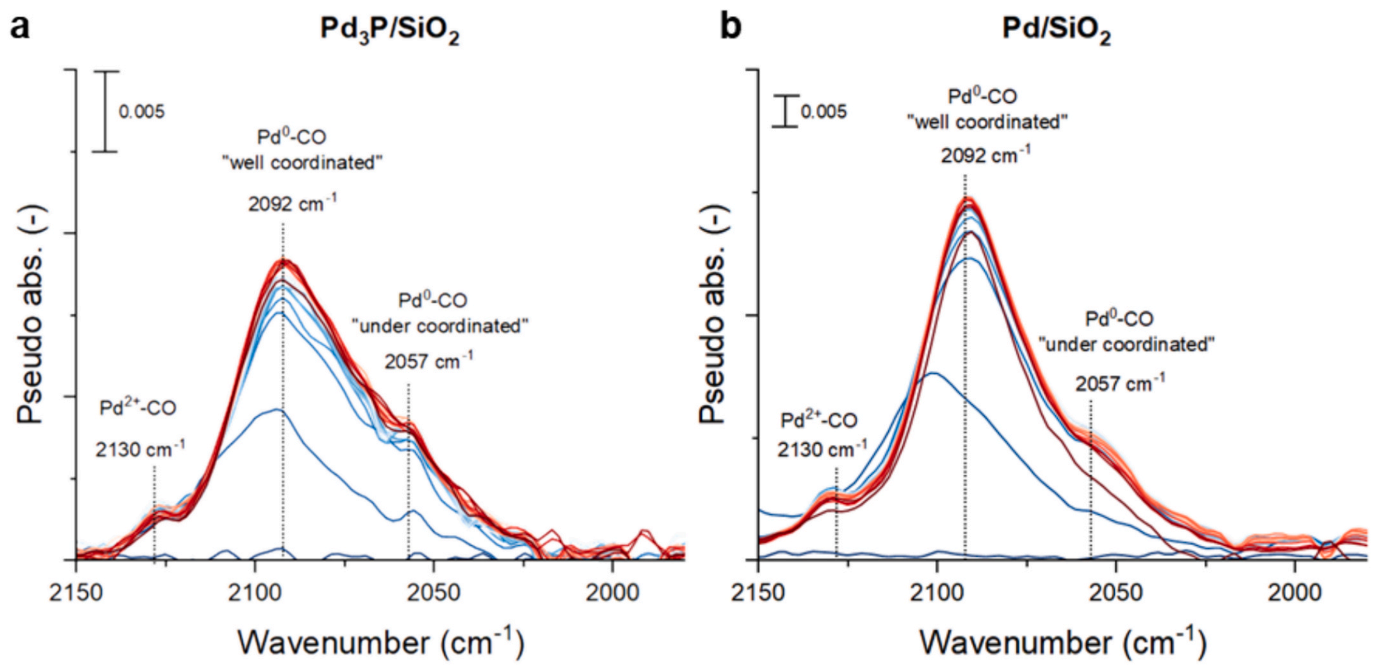

Fig. 4. CO-DRIFT spectra of a) Pd_3P/SiO_2 (5 wt% Pd) and b) Pd/SiO_2 (5 wt% Pd) conducted at 30 °C. Blue: beginning of the adsorption, red: last scan. The granulated catalysts were placed in a high-temperature *in-situ* cell and pre-treated for 1 h in argon at 150 °C to remove physically adsorbed water prior to the CO-DRIFT measurements. (For interpretation of the references to colour in this figure legend, the reader is referred to the web version of this article.)

To investigate the impact of phosphorus on the catalyst, the synthesized palladium phosphide catalysts and the reference system, Pd/SiO_2 (5 wt% Pd), were further investigated in the alkoxycarbonylation of iodobenzene. Clearly, Pd_3P/SiO_2 (5 wt% Pd) exhibited a significantly higher activity than Pd/SiO_2 (5 wt% Pd), demonstrating the impact of the d-metal-p-block element combination strategy (Table 1 and S1). A turn over frequency (TOF), calculated based on dispersion measurement *via* CO chemisorption, of 7600 h^{-1} was determined for the alkoxycarbonylation of iodobenzene at 100 °C with Pd_3P/SiO_2 (5 wt% Pd), whereas the application of Pd/SiO_2 (5 wt% Pd) resulted in a substantially lower TOF of 2800 h^{-1} (Table S1). This is remarkable, since the CO dispersion measurements have initially shown a higher dispersion of the palladium-based reference catalyst. Similar results were observed at higher levels of conversions. An iodobenzene conversion of 80 % (Table 1, entry 7) was observed after 1 h reaction time with Pd_3P/SiO_2 (5 wt% Pd), while only 50 % conversion (Table 1, entry 8) could be achieved with the reference catalysts Pd/SiO_2 (5 wt% Pd).

The significant enhancement in catalytic activity observed for Pd_3P/SiO_2 (5 wt% Pd) compared to Pd/SiO_2 (5 wt% Pd) strongly suggests that the activity is related to the unique crystal structure and surface arrangement of the palladium phosphide. Whereas neighboring Pd sites are observed in the crystal structure of pure palladium, the incorporation of phosphorus separates these contiguous sites, which results in the formation of single active sites in palladium phosphide. An analysis of lower miller index surfaces of Pd_3P, reveals interatomic distances of neighboring Pd atoms ranging from 2.78 Å to 3.24 Å in Pd_3P (100), up to 2.93 Å in Pd_3P (001) until 3.11 Å in Pd_3P (010), all of which are longer in comparison to the uniform Pd–Pd distance of 2.75 Å in elemental Pd

Table 1
Alkoxycarbonylation reaction of iodobenzene with various bases and catalysts performed in batch mode.

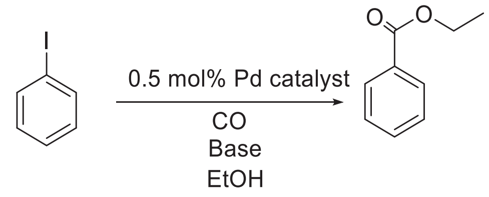

Entry	Catalyst	Base	Reaction time (h)	Iodobenzeneconversion (%)	Selectivity to ethyl benzoate(%)
1	Pd_3P/SiO_2 (5 wt% Pd)	Et_3N	2	97	100
2	Pd_3P/SiO_2 (5 wt% Pd)	Et_3N	3	100	100
3	Pd_3P/SiO_2 (5 wt% Pd)	NaOAc	2	40	100
4	Pd_3P/SiO_2 (5 wt% Pd)	KOH	2	70	98*
5	Pd_3P/SiO_2 (5 wt% Pd)	K_2CO_3	2	56	94*
6	Pd_3P/SiO_2 (5 wt% Pd)	–	2	7	100
7	Pd_3P/SiO_2 (5 wt% Pd)	Et_3N	1	80	100
8	Pd/SiO_2 (5 wt% Pd)	Et_3N	1	50	100

Reaction conditions: iodobenzene (1 mmol), base (3 mmol), CO (6 bar), ethanol (4 mL), catalyst (Pd 0.5 mol %), 100 °C, 1000 rpm. *Small amounts of benzene were observed.

[47]. Furthermore, the surface structures indicate the formation of various isolated Pd ensembles, which may act as potential active sites (Fig. 5). The site isolation phenomenon in Pd_3P, combined with the increased Pd interatomic distances, suggests that the oxidative addition of the organic substrate, which is the crucial first step in the catalytic cycle [63], will be more favorable for bulkier aromatic halides on isolated Pd sites, which are available on Pd_3P surfaces. This is attributed to a reduced steric hindrance, which significantly enhances catalytic activity. Additionally, the electronic effects induced by phosphorus [42] play a crucial role in optimizing the binding energy of substrates and intermediates on the active sites. These combined factors, namely the tailored steric and electronic properties, contribute to the exceptional performance of the Pd_3P/SiO_2 (5 wt% Pd) catalyst.

The TOF of Pd_3P/SiO_2 (5 wt% Pd) decreased markedly from 7600 h^{-1} at 6 bar CO to 820 h^{-1} at 25 bar CO (Table S1, entry 3), indicating a strong inhibitory effect of an excess of CO, which most likely stems from the formation of surface metal carbonyls [12,21]. This observation suggests that a high CO affinity can lead to catalyst poisoning, and a subsequent suppression of catalytic activity. To further probe this effect, the CO binding energies were estimated for Pd_3P surfaces using the Open Catalyst Project [66,67]. The calculated adsorption energies for CO on Pd_3P (100), (101), and (001) surfaces were −1.17 eV, −1.29 eV, and −1.23 eV, respectively. In contrast, the CO adsorption on conventional Pd (110) and Pd (111) surfaces [34] yielded significantly stronger binding energies of −1.72 eV and −1.74 eV, respectively. The reduced CO binding strength at Pd_3P surfaces is attributed to the electron transfer from Pd to P in the phosphide lattice, which decreases the electron density at the Pd centers, thereby weakening the π^* back-donation to CO. As a result, the formation of strongly bound carbonyl species is suppressed, minimizing surface poisoning and preserving active sites for the oxidative addition of aryl halides. These findings align with CO-DRIFTS and chemisorption data, both of which indicated a diminished CO adsorption on Pd_3P. Moreover, XPS analysis confirms the cationic character of Pd on the Pd_3P surface, resulting in the aforementioned weakening. Beyond the reduced CO affinity, CO-DRIFTS analysis revealed a higher proportion of undercoordinated Pd sites, such as edges and step sites, on Pd_3P/SiO_2 compared to Pd/SiO_2. These undercoordinated sites are known to facilitate oxidative addition processes [68], providing a plausible explanation for the enhanced alkoxycarbonylation activity observed with the Pd_3P catalyst. Although key material characteristics identified in this work, such as charge transfer effects, spatially separated undercoordinated Pd sites, and variations in CO affinity, have been clearly identified from the conducted measurements, the catalyst surface may still undergo restructuring under reaction conditions, potentially leading to deviations from the *ex-situ*–probed surface state. More definitive insights would therefore require *in-situ* or *operando* measurements, or catalyst pretreatment procedures under reaction conditions prior to analysis.

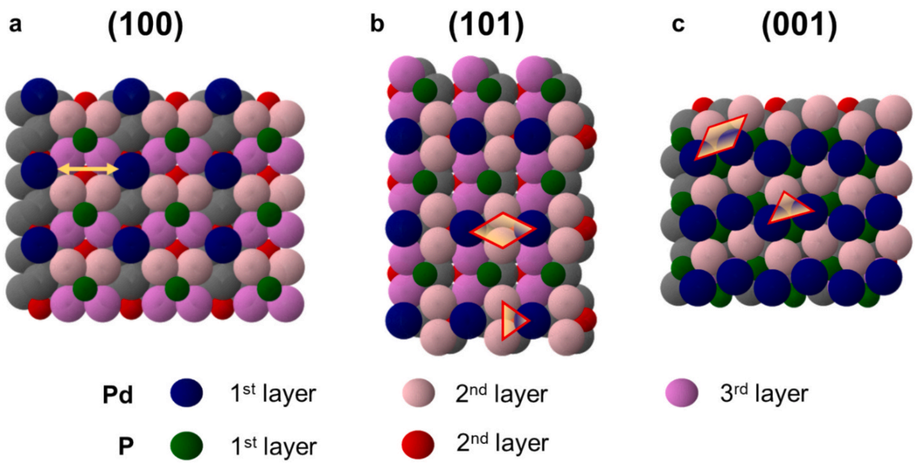

Fig. 5. Surfaces of Pd_3P with potential structural motifs and separation of the potential active sites. Images were generated with crystal data from the Material Project (ID: mp-19879).[65].

To further improve the catalytic activity of Pd_3P, the effect of reducing its particle size was investigated by employing a lower Pd loading of 1 wt%. PXRD measurements provided the clear evidence of crystallite size reduction, as indicated by the absence of distinct reflexes (Fig. S2). Surprisingly, the use of Pd_3P/SiO_2 (1 wt% Pd) as catalyst showed a negative impact on the catalytic activity, as a substantial reduction in conversion was observed in comparison to Pd_3P/SiO_2 (5 wt % Pd), despite both systems providing the same overall Pd concentration in the reaction mixture. The alkoxycarbonylation of iodobenzene with ethanol yielded a conversion of only 41 % (Table S2, entry 1) after 1 h at 100 °C and a CO pressure of 6 bar using Pd_3P/SiO_2 (1 wt% Pd). In contrast, a significantly higher conversion of 80 % (Table S2, entry 2) was achieved under the same reaction conditions with Pd_3P/SiO_2 (5 wt % Pd). The decline in activity, when using lower palladium phosphide loadings, may be attributed to surface poisoning effects, which are most likely caused by the strong binding of substrates or intermediates to the extremely small nanoparticles of the Pd_3P/SiO_2 (1 wt% Pd) catalyst.

To further investigate the effect of the Pd loading, Pd_3P/SiO_2 with a higher Pd content (10 wt%) was synthesized. PXRD analysis confirmed the exclusive formation of the Pd_3P phase, with sharper and less broadened reflections than those of the lower metal-loaded samples, which is indicative for an increased crystallite size (Fig. S3). The catalytic testing showed a higher activity for Pd_3P/SiO_2 (10 wt% Pd) compared to the catalysts with 5 and 1 wt% Pd loading, achieving 63 % iodobenzene conversion *versus* 55 % for the 5 wt% catalyst under identical reaction conditions (Table S2). These results at first glance indicate a positive influence of a higher Pd loading, as well as larger particle size or increased crystallinity, on the catalytic activity. Although the Pd_3P/SiO_2 (10 wt% Pd) catalyst exhibits enhanced activity, subsequent investigations were conducted using Pd_3P/SiO_2 (5 wt% Pd) in order to reduce the precious metal loading on the support, while maintaining high catalytic performance.

The kinetics of heterogeneous alkoxycarbonylation reactions have been largely unexplored in many literature-reported systems. To address

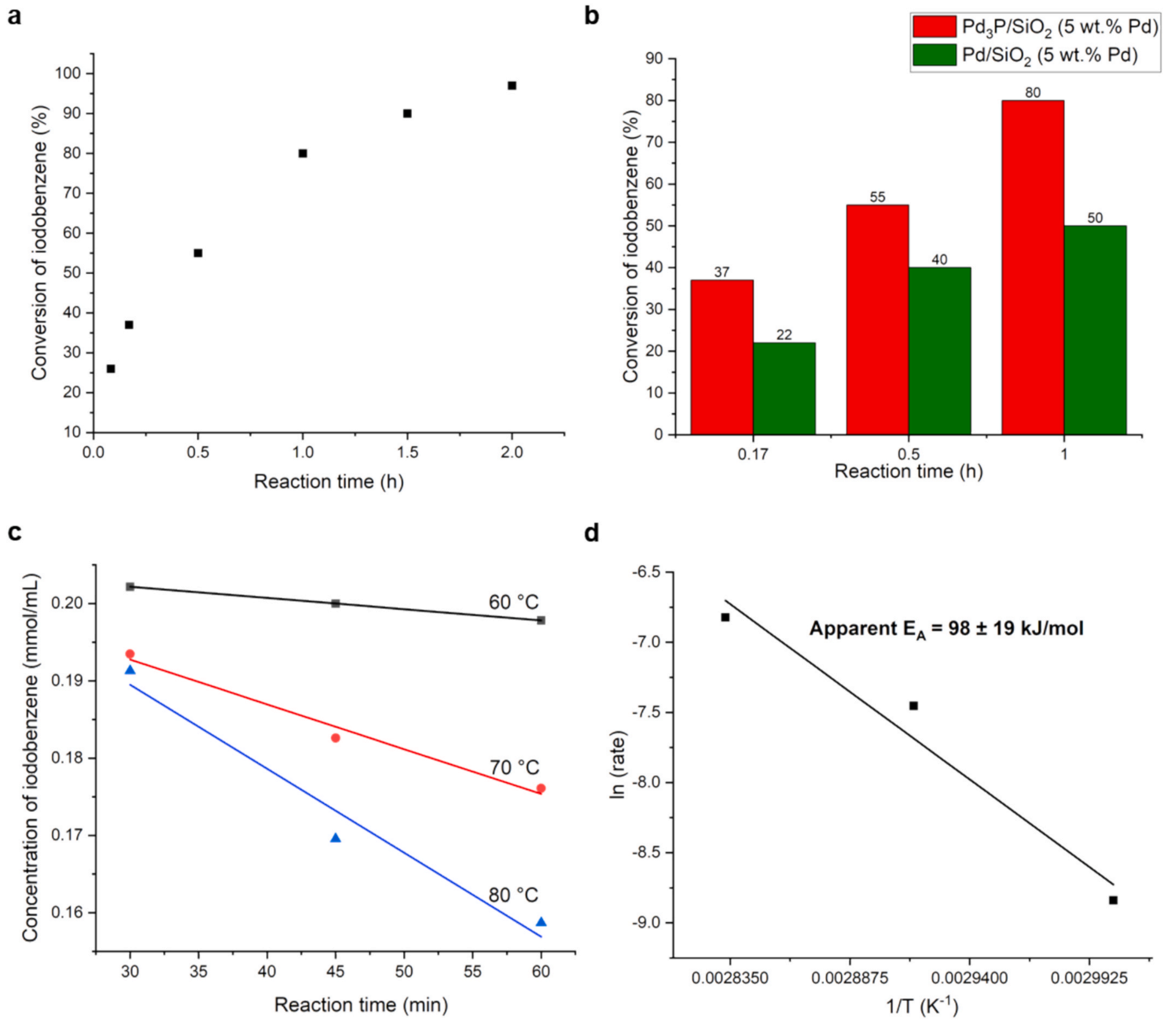

Fig. 6. a) Conversion data for the alkoxycarbonylation of iodobenzene using Pd_3P/SiO_2 (5 wt% Pd) and b) activity comparison with Pd/SiO_2 (5 wt% Pd). Reaction conditions: iodobenzene (1 mmol), Et_3N (3 mmol), CO (6 bar), ethanol (4 mL), catalyst (Pd 0.5 mol %), 100 °C, 1000 rpm. c) Determination of the reaction rate at various temperatures and d) Arrhenius plot for the estimation of the apparent activation energy of the Pd_3P/SiO_2 (5 wt% Pd) catalyzed alkoxycarbonylation of iodobenzene. Reaction conditions: iodobenzene (1 mmol), Et_3N (3 mmol) CO (6 bar), ethanol (4 mL), catalyst (Pd 0.5 mol %), 1000 rpm.

this gap, an investigation was conducted to determine the apparent activation energy of the Pd_3P/SiO_2 (5 wt% Pd) catalyzed alkoxycarbonylation of iodobenzene (Fig. 6). The experiments were performed in the temperature range of 60–80 °C and the apparent activation energy was estimated from the Arrhenius plot (ln (rate) v/s 1/T) to amount 98 ± 19 kJ/mol (Fig. 6d). The estimated apparent activation energy is comparable with the value reported by Ito *et al.* (104 kJ/mol) for the alkoxycarbonylation of iodobenzene with palladium black [69], whereby the underlying Arrhenius plot is based on rate constants and a higher temperature range (70–100 °C).

For a more in-depth comparison, the activities of homogeneous catalysts, such as palladium acetate ($Pd(OAc)_2$), which is a generally used catalyst precursor for homogeneous carbonylation reaction [21], and palladium nitrate ($Pd(NO_3)_2{\cdot}2H_2O$), were explored in the alkoxycarbonylation of iodobenzene and turn over frequencies were estimated. The TOF values of $Pd(OAc)_2$ (618 h^{-1}) and $Pd(NO_3)_3{\cdot}2H_2O$ (698 h^{-1}) were found to be significantly lower compared to those of Pd_3P/SiO_2 (5 wt% Pd), which exhibited a TOF of 7600 h^{-1} under identical reaction conditions (Table S3). These findings again underline the superior catalytic performance of the synthesized palladium phosphide catalyst. To further benchmark the performance of the synthesized catalysts against previously reported systems, a comparison with other heterogeneous catalysts was carried out based on the conversion of iodobenzene under comparable reaction conditions (Table S4). The results indicate that the Pd_3P/SiO_2 (5 wt% Pd) catalyst exhibits performance comparable to, and in some cases exceeding, that of reported heterogeneous systems.

3.2.1. Substrate and reaction scope

After the successful application of Pd_3P/SiO_2 (5 wt% Pd) in the alkoxycarbonylation of iodobenzene with ethanol, other alcohols, such as methanol and isopropanol, were explored as NuH and conversions of 97 and 96 % were observed, respectively (Table S5). Further, the substrate scope was extended towards substituted aryl halides, which resulted in excellent conversions and selectivity's ranging from 98 to 100 % for the ethoxycarbonylation of *p*-iodotoluene, *p*-bromo-iodobenzene and *p*-iodoanisole (Table S5), all of which are representatives of lignin-derived synthons. Unfortunately, no conversion was observed with the less reactive chlorobenzene substrate when the reaction was conducted at 100 °C under 6 bar of CO pressure (Table S5, entry 7). This outcome can be attributed to the less facile oxidative addition of this non-activated aryl halide to Pd_3P/SiO_2 (5 wt% Pd) [70].

Further, the applicability of the catalyst towards various carbonylation reactions were assessed considering amino and phenoxycarbonylation reactions. Aminocarbonylation reactions were performed in toluene as solvent with iodobenzene and aniline as substrates in the temperature range of 100 to 140 °C and 10 bar CO pressure. At 100 °C, iodobenzene showed only 10 % conversion to benzanilide after 5 h, whereas increasing the temperature to 140 °C significantly improved the conversion to 78 % (Table S6). A preliminary test conducted for the phenoxycarbonylation of iodobenzene in toluene as solvent using Pd_3P/SiO_2 (5 wt% Pd) as catalyst at 100 °C for 7 h resulted in 30 % conversion to phenyl benzoate (Table S5, entry 8). These results highlight the remarkable versatility of the synthesized Pd_3P/SiO_2 (5 wt% Pd) catalyst, demonstrating its exceptional performance in catalyzing a broad range of carbonylation reactions with various substituted aryl iodides and alcohols (NuH).

3.2.2. Recyclability studies

To assess the reusability of Pd_3P/SiO_2 (5 wt% Pd), the catalyst was recovered after the reaction (first run) by centrifugation and again used for the aloxycarboylation of iodobenzene. No decline in activity was observed in the first round of recycling giving 97 % conversion after 2 h. However, a substantial reduction of conversion to 50 % was observed in the second round of recycling (Table S7). The palladium content in the recovered catalyst from the first run was quantified *via* ICP-AES analysis to estimate the precious metal loss during the process. A reduction of palladium loading from 4.94 to 3.70 wt% was determined, which indicates a severe loss of Pd_3P nanoparticles from the support due to applied reaction conditions and mechanical abrasion induced by the fast-rotating stirrer in the batch autoclave. The observed Pd loss might also suggest that the reaction potentially proceeds, at least in part, *via* a homogeneous pathway involving soluble Pd species. A widely discussed leaching–redeposition mechanism in Pd-catalyzed reactions of aryl halides may therefore be operative [71,72], in which Pd species leach from the solid catalyst, catalyze the reaction in the liquid phase, and subsequently (partly or completely) redeposit on the support. Owing to the facile oxidative addition of aryl halides on Pd_3P, soluble Pd species may form more readily than on metallic Pd, enabling their transfer into the liquid phase. Oxidative addition is a key step governing Pd leaching, and the extent of this process is strongly influenced by reaction parameters, such as solvent, base, temperature, atmosphere, substrate, and the degree of surface disorder [68,73–75]. For instance, organic bases such as triethylamine can act as ligands, stabilizing soluble Pd complexes, and therefore lead to a shift from surface-mediated catalysis to a homogeneous mechanism [72,76]. Similarly, solvent polarity plays a critical role in determining the extent of leaching of Pd species from a catalyst surface [77]. In parallel, the thermal stability of the resulting molecular Pd species under reaction conditions also influences catalytic performance [75]. Taken together, these factors underscore the complexity of the alkoxycarbonylation reaction mechanism, for which the true nature of the active species, homogeneous versus heterogeneous, remains under debate. Definitive mechanistic conclusions will require a complex interplay of *in-situ* and/or *operando* studies, such as X-ray absorption spectroscopy and DFT calculations [68,78–80].

The HAADF-STEM analysis of the recovered Pd_3P/SiO_2 (5 wt% Pd) catalysts showed an increase in the particle size from 5.6 nm to 7.0 nm after the first run, revealing the sintering of nanoparticles under reaction conditions (Fig. S5). Such aggregation of palladium nanoparticles, facilitated by CO is a well-known phenomenon [5,22], and can therefore be considered as the reason for the increased particle size in the recovered catalyst, which ultimately leads to a subsequent decline in catalytic activity. The observed particle growth might also indicate the dissolution and redeposition of Pd species under reaction conditions, highlighting the need for further *in-situ* or *operando* investigations [78–80]. However, the structural integrity of the recovered catalyst was confirmed through HRTEM lattice fringe-based assessment (Fig. S5), were the d spacing of 2.0 Å corresponding to Pd_3P (122) indicates no phase transition or decomposition during catalysis. Leaching of Pd in heterogeneously catalyzed alkoxycarbonylation reactions has been reported, with various reasons postulated, eventually leading to the loss of catalytic activity [5]. Notably, a Pd loss of 25 % from the catalyst after alkoxycarbonylation with Pd/C was reported by Davies *et al.* [81], while Papp and co-workers [82] observed a 24 % loss during aminocarbonylation reactions with a Pd-containing supported ionic liquid phase catalyst. These findings highlight the significance of enhancing metal-support interactions in carbonylation catalysis. To stabilize the palladium phosphide and suppress metal leaching, other support materials, such as phosphorus-modified silica, which led to a substantial reduction in Pd leaching in Heck coupling reactions [44], modified carbon or ceria, or metal organic frameworks as supports, can be investigated. Moreover, a systematic selection of reaction conditions, such as solvent, temperature, CO pressure, and base may offer an effective approach to suppress Pd leaching and enhance the catalyst stability. In addition, the incorporation of suitable protectants into the reaction system represents another promising strategy to mitigate catalyst leaching and improve the recyclability of the catalyst.

4. Conclusions

The application of element synergy, which involves the incorporation of a non-metal from the p-block into a metal matrix and the

subsequent formation of mixed d-metal-p-block element phases, represents a very promising design strategy for heterogeneous catalysts. This approach has now been successfully extended to the field of carbonylation reactions, including alkoxycarbonylations, aminocarbonylations, and phenoxycarbonylations. In this study, the incorporation of phosphorus into palladium led to the formation of highly active supported Pd_3P nanoparticles, which outperformed conventionally supported Pd catalysts and even surpassed the activity of several homogeneous systems. The exceptional catalytic performance of Pd_3P/SiO_2 can be attributed to two main factors: the formation of well-defined, isolated active palladium sites and the electronic modulation induced by phosphorus, which acts similarly to a ligand by tuning the metal's electronic and geometric properties. Importantly, the use of aryl iodides as lignin-derived renewable synthons in carbonylation reactions highlights a sustainable pathway for converting biomass-derived aromatics into valuable chemical building blocks. This work not only demonstrates the potential of element synergy for the rational design of efficient heterogeneous catalysts but also contributes to advancing sustainable carbonylation chemistry.

5. Associated content

The Supporting Information is available free of charge and as open files on the NFDI4Cat Central Data Repository Repo4Cat (https://hdl.handle.net/21.11165/4cat/arp5-s69r). A pre-print was published on ChemRxiv (https://doi.org/10.26434/chemrxiv-2025-46zsr).

CRediT authorship contribution statement

Arjun Neyyathala: Writing – original draft, Investigation, Data curation. **Felix Jung:** Methodology, Investigation. **Claus Feldmann:** Writing – review & editing, Validation. **Simon Barth:** Methodology, Investigation. **Jan-Dierk Grunwaldt:** Writing – review & editing, Validation. **Ivana Jevtovikj:** Methodology, Investigation. **Stephan A. Schunk:** Writing – review & editing, Supervision, Conceptualization. **Paolo Dolcet:** Methodology, Investigation. **Silvia Gross:** Methodology, Investigation. **Schirin Hanf:** Writing – review & editing, Writing – original draft, Supervision, Methodology, Conceptualization.

Declaration of competing interest

The authors declare that they have no known competing financial interests or personal relationships that could have appeared to influence the work reported in this paper.

Acknowledgements

The authors gratefully acknowledge the funding for A.N. from BASF SE and the productive discussions with the team at hte GmbH. The authors would like to thank Ms. Kathrin Schaefer (ITCP, KIT) for performing CO chemisorption measurements. This study was also supported by the Deutsche Forschungsgemeinschaft (DFG, German Research Foundation) -SFB 1441- Project-ID 426888090. We also would like to express our gratitude to the NFDI4Cat community, which is funded by the Deutsche Forschungsgemeinschaft (DFG, German Research Foundation) - Project-ID 441926934, for valuable discussions. Silvia Gross gratefully thanks DFG for a Mercator Fellowship within the SFB 1441.

Appendix A. Supplementary data

Supplementary data to this article can be found online at https://doi.org/10.1016/j.jcat.2026.116733.

Data availability

The Supporting Information is available free of charge and as open files on the NFDI4Cat Central Data Repository Repo4Cat (https://hdl.handle.net/21.11165/4cat/arp5-s69r).

References

[1] H. Li, A. Bunrit, N. Li, F. Wang, Heteroatom-participated lignin cleavage to functionalized aromatics, Chem. Soc. Rev. 49 (12) (2020) 3748–3763, https://doi.org/10.1039/D0CS00078G.
[2] X. Wu, X. Fan, S. Xie, J. Lin, J. Cheng, Q. Zhang, L. Chen, Y. Wang, Solar energy-driven lignin-first approach to full utilization of lignocellulosic biomass under mild conditions, Nat. Catal. 1 (10) (2018) 772–780, https://doi.org/10.1038/s41929-018-0148-8.
[3] F. Brienza, D. Cannella, D. Montesdeoca, I. Cybulska, D.P. Debecker, A guide to lignin valorization in biorefineries: traditional, recent, and forthcoming approaches to convert raw lignocellulose into valuable materials and chemicals, RSC Sustain. 2 (1) (2024) 37–90, https://doi.org/10.1039/D3SU00140G.
[4] Y. Liu, Y. Li, Z. He, S. Wu, C. Ma, W. Li, S. Li, Z. Chen, S. Liu, B. Tian, Producing aryl halides from lignin, Nat. Commun. 16 (1) (2025) 3673, https://doi.org/10.1038/s41467-025-59054-0.
[5] B. Urbán, M. Papp, R. Skoda-Földes, Carbonylation of aryl halides in the presence of heterogeneous catalysts, Curr. Green Chem. 6 (2) (2019) 78–95, https://doi.org/10.2174/2213346106666190321141550.
[6] J.M. Tukacs, B. Marton, E. Albert, I. Tóth, L.T. Mika, Palladium-catalyzed aryloxy- and alkoxycarbonylation of aromatic iodides in γ-valerolactone as bio-based solvent, J. Organomet. Chem. 923 (2020) 121407, https://doi.org/10.1016/j.jorganchem.2020.121407.
[7] A. Brennführer, H. Neumann, M. Beller, Palladium-catalyzed carbonylation reactions of aryl halides and related compounds, Angew. Chem. Int. Ed. 48 (23) (2009) 4114–4133, https://doi.org/10.1002/anie.200900013.
[8] M.V. Khedkar, T. Sasaki, B.M. Bhanage, Immobilized palladium metal-containing ionic liquid-catalyzed alkoxycarbonylation, phenoxycarbonylation, and aminocarbonylation reactions, ACS Catal. 3 (3) (2013) 287–293, https://doi.org/10.1021/cs300719r.
[9] F. Messa, A.N. Paparella, D. Veselý, J. Krajčovič, P. Papadia, S. Perrone, A. Salomone, Gas-free amino- and alkoxycarbonylation of aryl iodides in a bioinspired deep eutectic solvent with Mo(CO)6 as a safe CO source, European J. Org. Chem. 26 (24) (2023) e202300309, https://doi.org/10.1002/ejoc.202300309.
[10] C.F.J. Barnard, Carbonylation of aryl halides: extending the scope of the reaction, Org. Process Res. Dev. 12 (4) (2008) 566–574, https://doi.org/10.1021/op800069w.
[11] A. Schoenberg, I. Bartoletti, R.F. Heck, Palladium-catalyzed carboalkoxylation of aryl, benzyl, and vinylic halides, J. Org. Chem. 39 (23) (1974) 3318–3326, https://doi.org/10.1021/jo00937a003.
[12] W. Mägerlein, M. Beller, A.F. Indolese, Palladium-catalyzed carbonylation of aryl halides — a detailed investigation of the alkoxycarbonylation of 4-bromoacetophenone, J. Mol. Catal. A Chem. 156 (1–2) (2000) 213–221, https://doi.org/10.1016/S1381-1169(99)00400-8.
[13] A. Yamamoto, F. Ozawa, N. Kawasaki, H. Okamoto, T. Yamamoto, Mechanisms of double and single carbonylation reactions of aryl iodides catalyzed by palladium complexes to give A-keto esters and esters, Organometallics 6 (8) (1987) 1640–1651, https://doi.org/10.1021/om00151a008.
[14] B. Apanda, S. Zolezzi, G. Valdebenito, J. Cáceres-vásquez, S.A. Moya, P. Aguirre, Aminocarbonylation reaction using palladium complexes containing phosphorus-nitrogen ligands as catalysts, J. Chil. Chem. Soc. 58 (4) (2013) 2136–2137, https://doi.org/10.4067/S0717-97072013000400052.
[15] E. Mizushima, T. Hayashi, M. Tanaka, Palladium-catalysed carbonylation of aryl halides in ionic liquid media: high catalyst stability and significant rate-enhancement in alkoxycarbonylation, Green Chem. 3 (2) (2001) 76–79, https://doi.org/10.1039/b100951f.
[16] H. Neumann, A. Brennführer, P. Groß, T. Riermeier, J. Almena, M. Beller, Efficient carbonylation of aryl and heteroaryl bromides using a palladium/diadamantylbutylphosphine catalyst, Adv. Synth. Catal. 348 (10–11) (2006) 1255–1261, https://doi.org/10.1002/adsc.200606044.
[17] J.R. Martinelli, T.P. Clark, D.A. Watson, R.H. Munday, S.L. Buchwald, Palladium-catalyzed aminocarbonylation of aryl chlorides at atmospheric pressure: the dual role of sodium phenoxide, Angew. Chem Int. Ed. 46 (44) (2007) 8460–8463, https://doi.org/10.1002/anie.200702943.
[18] T. Xu, H. Alper, Pd-catalyzed chemoselective carbonylation of aminophenols with iodoarenes: alkoxycarbonylation vs aminocarbonylation, J. Am. Chem. Soc. 136 (49) (2014) 16970–16973, https://doi.org/10.1021/ja508588b.
[19] A. Mata, C.A. Hone, B. Gutmann, L. Moens, C.O. Kappe, Continuous-flow Pd-catalyzed carbonylation of aryl chlorides with carbon monoxide at elevated temperature and pressure, ChemCatChem 11 (3) (2019) 997–1001, https://doi.org/10.1002/cctc.201801974.
[20] J.R. Martinelli, D.A. Watson, D.M.M. Freckmann, T.E. Barder, S.L. Buchwald, Palladium-catalyzed carbonylation reactions of aryl bromides at atmospheric pressure: a general system based on Xantphos, J. Org. Chem. 73 (18) (2008) 7102–7107, https://doi.org/10.1021/jo801279r.

[21] C.F.J. Barnard, Palladium-catalyzed carbonylation—A reaction come of age, Organometallics 27 (21) (2008) 5402–5422, https://doi.org/10.1021/om800549q.

[22] V. Dufaud, J. Thivolle-Cazat, J.-M. Basset, Palladium catalysed carbonylation of aryl chlorides to the corresponding methyl esters, J. Chem. Soc. Chem. Commun. 5 (1990) 426–427, https://doi.org/10.1039/c39900000426.

[23] J. Liu, J. Chen, C. Xia, A simple and efficient recyclable phosphine-free catalytic system for alkoxycarbonylation and carbonylative sonogashira coupling reactions of aryl iodides, J. Catal. 253 (1) (2008) 50–56, https://doi.org/10.1016/j.jcat.2007.10.021.

[24] F. Chen, T. Li, X. Pan, Y. Guo, B. Han, F. Liu, B. Qiao, A. Wang, T. Zhang, Pd1/CeO2 single-atom catalyst for alkoxycarbonylation of aryl iodides, Sci. China Mater. 63 (6) (2020) 959–964, https://doi.org/10.1007/s40843-019-1204-y.

[25] A. Siva Prasad, B. Satyanarayana, Fe3O4 supported Pd(0) nanoparticles catalyzed alkoxycarbonylation of aryl halides, J. Mol. Catal. A Chem. 370 (2013) 205–209, https://doi.org/10.1016/j.molcata.2013.01.002.

[26] I. Ziccarelli, H. Neumann, C. Kreyenschulte, B. Gabriele, M. Beller, Pd-supported on N-doped carbon: improved heterogeneous catalyst for base-free alkoxycarbonylation of aryl iodides, Chem. Commun. 52 (86) (2016) 12729–12732, https://doi.org/10.1039/c6cc07269k.

[27] Y. Zhang, Y. Xiong, J. Ge, R. Lin, C. Chen, Q. Peng, D. Wang, Y. Li, Porous organic cage stabilised palladium nanoparticles: efficient heterogeneous catalysts for carbonylation reaction of aryl halides, Chem. Commun. 54 (22) (2018) 2796–2799, https://doi.org/10.1039/c7cc09918e.

[28] H. Mei, S. Xiao, T. Zhu, Y. Lei, G. Li, Alkoxycarbonylation and phenoxycarbonylation reactions catalyzed by a Palladium(II) organometallic complex encaged in y zeolite, Transit. Met. Chem. 39 (4) (2014) 443–450, https://doi.org/10.1007/s11243-014-9818-9.

[29] M.B. Ibrahim, R. Suleiman, M. Fettouhi, B. El Ali, A palladium-bisoxazoline supported catalyst for selective synthesis of aryl esters and aryl amides: via carbonylative coupling reactions, RSC Adv. 6 (82) (2016) 78826–78837, https://doi.org/10.1039/c6ra15506e.

[30] V.V. Gaikwad, V.B. Saptal, K. Harada, T. Sasaki, D. Nishio-Hamane, B.M. Bhanage, Ionic liquid immobilized on graphene-oxide-containing palladium metal ions as an efficient catalyst for the alkoxy, amino, and phenoxy carbonylation reactions, ChemNanoMat 4 (6) (2018) 575–582, https://doi.org/10.1002/cnma.201700384.

[31] B. Liu, X. Lan, Q. Zhong, T. Wang, Metal phosphide: an atypical catalytic site, ACS Catal. 14 (2024) 757–775.

[32] S. Hanf, L.A. Rupflin, R. Gläser, S.A. Schunk, Current state of the art of the solid Rh-based catalyzed hydroformylation of short-chain olefins, Catalysts 10 (5) (2020) 1–36, https://doi.org/10.3390/catal10050510.

[33] L. Karam, C. Farès, C. Weidenthaler, C.N. Neumann, Expedited synthesis of metal phosphides maximizes dispersion, air stability, and catalytic performance in selective hydrogenation, Angew. Chem. Int. Ed. 63 (33) (2024) e202404292, https://doi.org/10.1002/anie.202404292.

[34] D. Albani, M. Shahrokhi, Z. Chen, S. Mitchell, R. Hauert, N. López, J. Pérez-Ramírez, Selective ensembles in supported palladium sulfide nanoparticles for alkyne semi-hydrogenation, Nat. Commun. 9 (1) (2018) 1–11, https://doi.org/10.1038/s41467-018-05052-4.

[35] A. Neyyathala, E. Fako, S. De, D. Gashnikova, F. Maurer, J.-D. Grunwaldt, S. A. Schunk, S. Hanf, From poison to promotor: spatially isolated metal sites in supported rhodium sulfides as hydroformylation catalysts, Small Str. 6 (2025) 2400260, https://doi.org/10.1002/sstr.202400260.

[36] L. Alvarado Rupflin, J. Mormul, M. Lejkowski, S. Titlbach, R. Papp, R. Gläser, M. Dimitrakopoulou, X. Huang, A. Trunschke, M.G. Willinger, R. Schlögl, F. Rosowski, S.A. Schunk, Platinum group metal phosphides as heterogeneous catalysts for the gas-phase hydroformylation of small olefins, ACS Catal. 7 (5) (2017) 3584–3590, https://doi.org/10.1021/acscatal.7b00499.

[37] A. Neyyathala, F. Flecken, S. Hanf, A supported palladium phosphide catalyst for the wacker-tsuji-oxidation of styrene, ChemPlusChem 88 (2) (2023) e202200431, https://doi.org/10.1002/CPLU.202200431.

[38] S. Vásquez-Céspedes, R.C. Betori, M.A. Cismesia, J.K. Kirsch, Q. Yang, Heterogeneous catalysis for cross-coupling reactions: an underutilized powerful and sustainable tool in the fine chemical industry? Org. Process Res. Dev. 25 (4) (2021) 740–753, https://doi.org/10.1021/ACS.OPRD.1C00041.

[39] J. Masud, T. Van Nguyen, N. Singh, E. McFarland, M. Ikenberry, K. Hohn, C.-J. Pan, B.-J. Hwang, A Rh x S y /C catalyst for the hydrogen oxidation and hydrogen evolution reactions in HBr, J. Electrochem. Soc. 162 (4) (2015) F455–F462, https://doi.org/10.1149/2.0901504jes.

[40] D. Albani, K. Karajovic, B. Tata, Q. Li, S. Mitchell, N. López, J. Pérez-Ramírez, Ensemble design in nickel phosphide catalysts for alkyne semi-hydrogenation, ChemCatChem 11 (1) (2019) 457–464, https://doi.org/10.1002/CCTC.201801430.

[41] E. Nishiwaki, P.S. Rice, D.-Y. Kuo, F.Y. Dou, A. Pyka, B. Reid, H.A. Nguyen, E. M. Stuve, S. Raugei, B.M. Cossairt, Ni2P active site ensembles tune electrocatalytic nitrate reduction selectivity, Chem. Commun. 60 (54) (2024) 6941–6944, https://doi.org/10.1039/D4CC01834F.

[42] B. Liu, N. Huang, Y. Wang, X. Lan, T. Wang, Promotion of inorganic phosphorus on rh catalysts in styrene hydroformylation: geometric and electronic effects, ACS Catal. 11 (3) (2021) 1787–1796, https://doi.org/10.1021/acscatal.0c04684.

[43] Y. Lee, W. Jeong, Y.J. Hwang, B. An, H. Lee, H. Jeong, G. Kim, Y. Park, M. Kim, D.-H. Ha, Basics, developments, and strategies of transition metal phosphides toward electrocatalytic water splitting: beyond noble metal catalysts, J. Mater. Chem. A 12 (42) (2024) 28574–28594, https://doi.org/10.1039/D4TA04455J.

[44] A. Neyyathala, F. Flecken, F. Rang, C. Papke, S. Hanf, Support engineering for the stabilisation of heterogeneous Pd3P-based catalysts for heck coupling reactions, Chem. Eur. J. 30 (2024) e202302825, https://doi.org/10.1002/chem.202302825.

[45] A.Y. Lee, C.J. Powell, J.M. Gorham, A. Morey, J.H.J. Scott, R.J. Hanisch, Development of the NIST X-ray photoelectron spectroscopy (XPS) database, version 5, Data Sci. J. 23 (2024) 45, https://doi.org/10.5334/dsj-2024-045.

[46] S. Carenco, Y. Hu, I. Florea, O. Ersen, C. Boissière, N. Mézailles, C. Sanchez, Metal-dependent interplay between crystallization and phosphorus diffusion during the synthesis of metal phosphide nanoparticles, Chem. Mater. 24 (21) (2012) 4134–4145, https://doi.org/10.1021/cm3022243.

[47] Y. Liu, A.J. McCue, C. Miao, J. Feng, D. Li, J.A. Anderson, Palladium phosphide nanoparticles as highly selective catalysts for the selective hydrogenation of acetylene, J. Catal. 364 (2018) 406–414, https://doi.org/10.1016/j.jcat.2018.06.001.

[48] S. Xing, M. He, G. Lv, F. Xu, F. Wang, H. Zhang, Y. Wang, Palladium phosphide nanoparticles embedded in 3D N, P Co-doped carbon film for high-efficiency oxygen reduction, J. Mater. Sci. 56 (17) (2021) 10523–10536, https://doi.org/10.1007/s10853-021-05935-w.

[49] A.K. Sharma, H. Joshi, R. Bhaskar, A.K. Singh, Solvent-tailored Pd3P0.95 nano catalyst for amide–nitrile inter-conversion, the hydration of nitriles and transfer hydrogenation of the C–O bond, Dalt. Trans. 48 (29) (2019) 10962–10970, https://doi.org/10.1039/C8DT04667K.

[50] J. Li, W. Chen, H. Zhao, X. Zheng, L. Wu, H. Pan, J. Zhu, Y. Chen, J. Lu, Size-dependent catalytic activity over carbon-supported palladium nanoparticles in dehydrogenation of formic acid, J. Catal. 352 (2017) 371–381, https://doi.org/10.1016/j.jcat.2017.06.007.

[51] Y. Soni, S. Pradhan, M.K. Bamnia, A.K. Yadav, S.N. Jha, D. Bhattacharyya, T. S. Khan, M.A. Haider, C.P. Vinod, Spectroscopic evidences for the size dependent generation of Pd species responsible for the low temperature CO oxidation activity on Pd-SBA-15 nanocatalyst, Appl. Catal. B Environ. 272 (2020) 118934, https://doi.org/10.1016/j.apcatb.2020.118934.

[52] L.S. Kibis, A.I. Stadnichenko, S.V. Koscheev, V.I. Zaikovskii, A.I. Boronin, Highly oxidized palladium nanoparticles comprising Pd 4+ species: spectroscopic and structural aspects, thermal stability, and reactivity, J. Phys. Chem. C 116 (36) (2012) 19342–19348, https://doi.org/10.1021/jp305166k.

[53] P. Massiot, M. Centeno, I. Carrizosa, J. Odriozola, Thermal evolution of sol–gel-obtained phosphosilicate solids (SiPO), J. Non Cryst. Solids 292 (1–3) (2001) 158–166, https://doi.org/10.1016/S0022-3093(01)00854-7.

[54] A.S. Ivanova, E.M. Slavinskaya, R.V. Gulyaev, V.I. Zaikovskii, O.A. Stonkus, I. G. Danilova, L.M. Plyasova, I.A. Polukhina, A.I. Boronin, Metal–support interactions in Pt/Al2O3 and Pd/Al2O3 catalysts for CO oxidation, Appl. Catal. B Environ. 97 (1–2) (2010) 57–71, https://doi.org/10.1016/j.apcatb.2010.03.024.

[55] J. Szanyi, J.H. Kwak, Dissecting the steps of CO2 reduction: 2. The interaction of CO and CO2 with Pd/γ-Al2O3: an in situ FTIR stud, Phys. Chem. Chem. Phys. 16 (29) (2014) 15126–15138, https://doi.org/10.1039/C4CP00617H.

[56] D.G. Oh, J. Lee, E. Kim, E.J. Jang, J.M. Kim, J.H. Kwak, Pd/SiO2 as an active and durable CH4 oxidation catalyst for vehicle applications, J. Ind. Eng. Chem. 99 (2021) 90–97, https://doi.org/10.1016/j.jiec.2021.04.012.

[57] D. Gun Oh, H.A. Aleksandrov, H. Kim, I.Z. Koleva, K. Khivantsev, G.N. Vayssilov, J. Hun Kwak, Key role of A-top CO on terrace sites of metallic Pd clusters for CO oxidation, Chem. Eur. J. 28 (49) (2022), https://doi.org/10.1002/chem.202200684.

[58] B.B. Sarma, J. Jelic, D. Neukum, D.E. Doronkin, X. Huang, F. Studt, J. D. Grunwaldt, Tracking and understanding dynamics of atoms and clusters of late transition metals with in-situ DRIFT and XAS spectroscopy assisted by DFT, J. Phys. Chem. C 127 (6) (2023) 3032–3046, https://doi.org/10.1021/acs.jpcc.2c07263.

[59] M. Zhao, Fabrication of ultrafine palladium phosphide nanoparticles as highly active catalyst for chemoselective hydrogenation of alkynes, Chem. Asian J. 11 (4) (2016) 461–464, https://doi.org/10.1002/asia.201500939.

[60] R.S. Mane, T. Sasaki, B.M. Bhanage, silica supported palladium-phosphine as a reusable catalyst for alkoxycarbonylation and aminocarbonylation of aryl and heteroaryl iodides, RSC Adv. 5 (115) (2015) 94776–94785, https://doi.org/10.1039/C5RA18692G.

[61] S.M. Islam, K. Ghosh, A.S. Roy, R.A. Molla, Polymer supported Pd catalyzed carbonylation of aryl bromides for the synthesis of aryl esters and amides, RSC Adv. 4 (73) (2014) 38986–38999, https://doi.org/10.1039/C4RA05365F.

[62] Y. Lei, L. Wu, X. Zhang, H. Mei, Y. Gu, G. Li, Palladium supported on triphenylphosphine functionalized porous organic polymer: a highly active and recyclable catalyst for alkoxycarbonylation of aryl iodides, J. Mol. Catal. A Chem. 398 (2015) 164–169, https://doi.org/10.1016/j.molcata.2014.12.008.

[63] P.E. Garrou, R.F. Heck, The mechanism of carbonylation of Halo (Bis Ligand) Organoplatinum (II), -Palladium (II), and Nickel(II) complexes, J. Am. Chem. Soc. 4115 (6) (1975) 4115–4127.

[64] Y. Hu, J. Liu, Z. Lu, X. Luo, H. Zhang, Y. Lan, A. Lei, Base-induced mechanistic variation in palladium-catalyzed carbonylation of aryl iodides, J. Am. Chem. Soc. 132 (9) (2010) 3153–3158, https://doi.org/10.1021/ja909962f.

[65] A. Jain, S.P. Ong, G. Hautier, W. Chen, W.D. Richards, S. Dacek, S. Cholia, D. Gunter, D. Skinner, G. Ceder, K.A. Persson, Commentary: the materials project: a materials genome approach to accelerating materials innovation, APL Mater. 1 (1) (2013) 11002, https://doi.org/10.1063/1.4812323/119685.

[66] L. Chanussot, A. Das, S. Goyal, T. Lavril, M. Shuaibi, M. Riviere, K. Tran, J. Heras-Domingo, C. Ho, W. Hu, A. Palizhati, A. Sriram, B. Wood, J. Yoon, D. Parikh, C. L. Zitnick, Z. Ulissi, Open catalyst 2020 (OC20) dataset and community challenges, ACS Catal. 11 (10) (2021) 6059–6072, https://doi.org/10.1021/acscatal.0c04525.

[67] J. Lan, A. Palizhati, M. Shuaibi, B.M. Wood, B. Wander, A. Das, M. Uyttendaele, C. L. Zitnick, Z.W. Ulissi, AdsorbML: A leap in efficiency for adsorption energy

calculations using generalizable machine learning potentials, npj Comput. Mater. 9 (1) (2023) 172, https://doi.org/10.1038/s41524-023-01121-5.
[68] M.V. Polynski, Y.S. Vlasova, Y.V. Solovev, S.M. Kozlov, V.P. Ananikov, Computational analysis of R–X oxidative addition to Pd nanoparticles, Chem. Sci. 15 (26) (2024) 9977–9986, https://doi.org/10.1039/D4SC00628C.
[69] T. Ito, K. Mori, T. Mizoroki, A. Ozaki, Effect of base on palladium-black catalyzed carbonylation of iodobenzene, Bull. Chem. Soc. Jpn 48 (7) (1975) 2091–2094, https://doi.org/10.1246/bcsj.48.2091.
[70] W. Mägerlein, A.F. Indolese, M. Beller, Development of new palladium catalysts for the alkoxycarbonylation of aryl chlorides, J. Organomet. Chem. 641 (1–2) (2002) 30–40, https://doi.org/10.1016/S0022-328X(01)01293-1.
[71] C. Gnad, A. Abram, A. Urstöger, F. Weigl, M. Schuster, K. Köhler, Leaching mechanism of different palladium surface species in heck reactions of aryl bromides and chlorides, ACS Catal. 10 (11) (2020) 6030–6041, https://doi.org/10.1021/acscatal.0c01166.
[72] M.V. Polynski, V.P. Ananikov, Modeling key pathways proposed for the formation and evolution of “cocktail”-type systems in Pd-catalyzed reactions involving ArX reagents, ACS Catal. 9 (5) (2019) 3991–4005, https://doi.org/10.1021/acscatal.9b00207.
[73] R.G. Heidenreich, J.G.E. Krauter, J. Pietsch, K. Köhler, Control of Pd leaching in heck reactions of bromoarenes catalyzed by Pd supported on activated carbon, J. Mol. Catal. A Chem. 182–183 (2002) 499–509, https://doi.org/10.1016/S1381-1169(01)00499-X.
[74] B.D. Briggs, N.M. Bedford, S. Seifert, H. Koerner, H. Ramezani-Dakhel, H. Heinz, R. R. Naik, A.I. Frenkel, M.R. Knecht, Atomic-scale identification of Pd leaching in nanoparticle catalyzed C–C coupling: effects of particle surface disorder, Chem. Sci. 6 (11) (2015) 6413–6419, https://doi.org/10.1039/C5SC01424G.
[75] S.S. Soomro, F.L. Ansari, K. Chatziapostolou, K. Köhler, Palladium leaching dependent on reaction parameters in Suzuki–Miyaura coupling reactions catalyzed by palladium supported on alumina under mild reaction conditions, J. Catal. 273 (2) (2010) 138–146, https://doi.org/10.1016/j.jcat.2010.05.007.
[76] F. Zhao, M. Shirai, Y. Ikushima, M. Arai, The leaching and re-deposition of metal species from and onto conventional supported palladium catalysts in the heck reaction of iodobenzene and methyl acrylate in N-methylpyrrolidone, J. Mol. Catal. A Chem. 180 (1–2) (2002) 211–219, https://doi.org/10.1016/S1381-1169(01)00436-8.
[77] I. Sádaba, M. López Granados, A. Riisager, E. Taarning, Deactivation of solid catalysts in liquid media: the case of leaching of active sites in biomass conversion reactions, Green Chem. 17 (8) (2015) 4133–4145, https://doi.org/10.1039/C5GC00804B.
[78] N. Yuan, V. Pascanu, Z. Huang, A. Valiente, N. Heidenreich, S. Leubner, A.K. Inge, J. Gaar, N. Stock, I. Persson, B. Martín-Matute, X. Zou, Probing the evolution of palladium species in Pd@MOF catalysts during the heck coupling reaction: an Operando X-ray absorption spectroscopy study, J. Am. Chem. Soc. 140 (26) (2018) 8206–8217, https://doi.org/10.1021/jacs.8b03505.
[79] S. Reimann, J. Stötzel, R. Frahm, W. Kleist, J.-D. Grunwaldt, A. Baiker, Identification of the active species generated from supported Pd catalysts in heck reactions: an in situ quick scanning EXAFS investigation, J. Am. Chem. Soc. 133 (11) (2011) 3921–3930, https://doi.org/10.1021/ja108636u.
[80] P.J. Ellis, I.J.S. Fairlamb, S.F.J. Hackett, K. Wilson, A.F. Lee, Evidence for the surface-catalyzed Suzuki–Miyaura reaction over palladium nanoparticles: an operando XAS study, Angew. Chemie Int. Ed. 49 (10) (2010) 1820–1824, https://doi.org/10.1002/anie.200906675.
[81] I.W. Davies, L. Matty, D.L. Hughes, P.J. Reider, Are heterogeneous catalysts precursors to homogeneous catalysts? J. Am. Chem. Soc. 123 (41) (2001) 10139–10140, https://doi.org/10.1021/ja016877v.
[82] M. Papp, R. Skoda-Földes, Phosphine-free double carbonylation of iodobenzene in the presence of reusable supported palladium catalysts, J. Mol. Catal. A Chem. 378 (2013) 193–199, https://doi.org/10.1016/j.molcata.2013.06.002.
